# Integration of metaheuristic based feature selection with ensemble representation learning models for privacy aware cyberattack detection in IoT environments

**DOI:** 10.1038/s41598-025-05545-5

**Published:** 2025-07-02

**Authors:** M. Karthikeyan, R. Brindha, Maria Manuel Vianny, V. Vaitheeshwaran, Mrinal Bachute, Sanket Mishra, Bibhuti Bhusan Dash

**Affiliations:** 1 Department of Computing Technologies, School of Computing, SRM Institute of Science and Technology, Chennai, 603203 India; 2 Department of Artificial Intelligence and Data Science Panimalar Engineering College, Chennai, 600123 India; 3 Department of Computer Science and Engineering, Aditya University, Surampalem, Kakinada India; 4 Department of Electronics and Telecommunications Engineering, Symbiosis Institute of Technology, Pune, India; 5 Manipal Institute of Technology Bengaluru, Manipal Academy of Higher Education, Manipal, India; 6 School of Computer Applications, KIIT Deemed to be University, Bhubaneswar, India

**Keywords:** Feature selection, Ensemble learning model, Cyberattack detection, Social group optimization, Data normalization, Computer science, Information technology

## Abstract

The Internet of Things (IoT) connects virtual and physical objects inserted with software, devices, and other technology that interchange data utilizing the Internet. It enables diverse devices and individuals to exchange data, interconnect, and personalize services to ease usage. Despite IoT’s merits, rising cyberthreats and the rapid growth of smart devices increase the risk of data breaches and security attacks. The increasing complexity of cyberattacks demands advanced intrusion detection systems (IDS) to defend crucial assets and data. AI techniques such as machine learning (ML) and deep learning (DL) have shown robust potential in improving IDS performance by accurately detecting and classifying malicious network behavior in IoT environments. This manuscript proposes an Adaptive Metaheuristic-Based Feature Selection with Ensemble Learning Model for Privacy-Preserving Cyberattack Detection (AMFS-ELPPCD) technique. The data normalization stage initially applies Z-score normalization to convert input data into a beneficial format. The AMFS-ELPPCD model utilizes the adaptive Harris hawk optimization (AHHO) model for the feature process selection of the subset. Furthermore, ensemble models such as bidirectional gated recurrent unit (BiGRU), Wasserstein autoencoder (WAE), and deep belief network (DBN) are used for the classification process. Finally, social group optimization (SGO) optimally adjusts the ensemble classifiers’ hyperparameter values, resulting in better classification performance. A set of simulations is performed to exhibit the promising results of the AMFS-ELPPCD under dual datasets. The experimental validation of the AMFS-ELPPCD technique portrayed a superior accuracy value of 99.44% and 98.85% under the CICIDS-2017 and NSLKDD datasets over existing models.

## Introduction

**Fig. 1:**
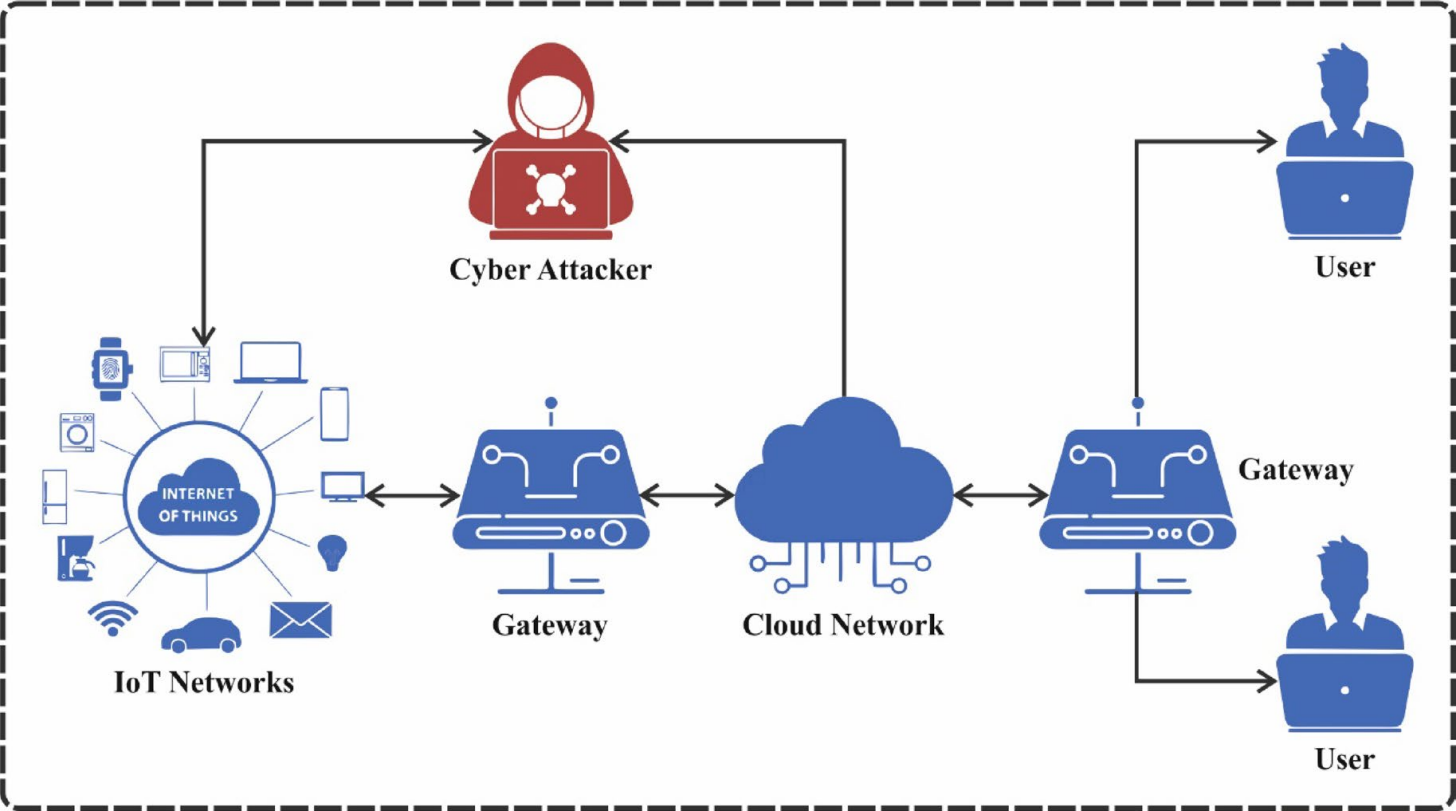
Cyberattack detection in IoT networks.

Recently, the IoT is experiencing considerable growth, with billions of devices connected globally^1^. The extensive acceptance of IoT devices has established many possibilities for improved effectiveness, competence, and user understanding^2^. Nevertheless, expanding IoT devices has additionally presented additional tasks to the cybersecurity environment. Cyberattacks on IoT devices can lead to severe impacts, like disruption of critical services, unauthorized access, and data breaches^3^. The terms IoT cybersecurity threats and cybercrime are numerous and inappropriately limited to the description of unethical activity, in which a system is a critical portion of the cybercrime and is frequently used to define normal crimes. IoT cybersecurity attacks are commonly determined as cybercriminal activity associated with IoT infrastructure^4^. The cyberattack difficulties, particularly those associated with hackers, infractions have become more prominent. Figure 1 illustrates the general structure of cyberattack detection in IoT networks. The hackers are computer programmers with advanced knowledge of computers and, for unethical purposes, frequently use such experience. To protect computer systems from assaults, IDSs are being established to help guarantee safer access and avoid privacy loss and user authentication^5^. An IDS initially gathers and handles data and then utilizes a recognition method to increase alarms, which are transferred to human networking experts for additional screening^2^. Deliberately, conventional IDSs might not be managed by standard IoT devices due to limited sources of such appliances^6^.

Therefore, the network-based IDS should be the exclusive method to execute the detection method for the IoT environment. Recent attack prediction technologies cannot follow the large number and form of attacks. Investigators have recently highlighted ML, predominantly DL models, because of their excellent outcomes in various prediction-based areas^7^. As concerns IoT, the usage of AI models like DL and ML techniques may provide an effective model for utilizing data to predict and recognize possible cybersecurity threats. As an approach to detect cyberattacks, the DL method is developing in popularity more quickly than preceding models that allow more effective mitigation^8^. DL is a subsection of AI that focuses on executing computing device applications that can identify composite, non-linear patterns and then use those designs to make predictions^9^. In the presence of privacy protection, DL methods have become progressively popular devices, and they have quickly become an essential element of current defence approaches against dangerous attacks. The widespread deployment of interconnected smart devices has significantly expanded the digital attack surface, increasing the vulnerability of IoT networks to advanced cyber threats. Ensuring intelligent and timely threat detection is crucial to safeguarding data integrity and maintaining secure operations in these dynamic environments.

This manuscript proposes an Adaptive Metaheuristic-Based Feature Selection with Ensemble Learning Model for Privacy-Preserving Cyberattack Detection (AMFS-ELPPCD) technique. The data normalization stage initially applies Z-score normalization to convert input data into a beneficial format. The AMFS-ELPPCD model utilizes the adaptive Harris hawk optimization (AHHO) model for the feature process selection of the subset. Furthermore, ensemble models such as bidirectional gated recurrent unit (BiGRU), Wasserstein autoencoder (WAE), and deep belief network (DBN) are used for the classification process. Finally, social group optimization (SGO) optimally adjusts the ensemble classifiers’ hyperparameter values, resulting in better classification performance. A set of simulations is performed to exhibit the promising results of the AMFS-ELPPCD approach under dual datasets. The key contribution of the AMFS-ELPPCD approach is listed below.

- The AMFS-ELPPCD model utilizes Z-score normalization to scale input features, ensuring consistency across varying data ranges. This enhances the learning process by stabilizing training and accelerating convergence. The technique also assists in generalizing across various IoT data dispersions.
- The AMFS-ELPPCD method employs the AHHO technique for effectual feature subset selection, minimizing dimensionality without losing critical data characteristics. This improves computational efficiency and model interpretability. The model also confirms that only the most relevant features are retained for precise classification.
- The AMFS-ELPPCD approach integrates an ensemble of BiGRU, WAE, and DBN techniques to integrate temporal dynamics and complex non-linear patterns in the data. This layered architecture improves detection accuracy and resilience against various cyberattack scenarios. The model also enables comprehensive feature learning for enhanced classification performance.
- The AMFS-ELPPCD methodology implements the SGO approach to fine-tune the ensemble framework’s hyperparameters. This optimization improves the model’s precision and adaptability across varying intrusion patterns, ensuring a balanced trade-off between accuracy and computational efficiency.
- The AMFS-ELPPCD technique highlights novelty in the unique combination of AHHO-based feature selection with a multi-model ensemble classifier and SGO-based hyperparameter tuning, presents a comprehensive and scalable methodology for accurate and resource-aware cyberattack detection in complex IoT environments.

## Review of literature

Alabdan et al.^10^ present a DL model, namely the Improved Crayfish Optimizer Approach with Interval Type-2 Fuzzy DL (ICOA-IT2FDL) approach for ID. BC technology is utilized for security. This approach employs the linear scaling normalization (LSN) method for normalizing the data. Additionally, an ICOA technique is implemented for feature selection. Moreover, the IT2 fuzzy DBN (IT2-FDBN) technique is employed for ID. Lastly, the bald eagle search (BES) approach enhances ID detection. Rbah et al.^11^ propose a fusion technique by integrating software-defined networking (SDN) with DL methods comprising NN, namely CNN and BiLSTM. This methodology also utilizes a unique integration enabling dynamic and effectual IoMT safety administration. The system handles various IoMTs by incorporating these techniques, providing an overall solution. Unlike conventional techniques, the fusion solution effortlessly adapts to the growing healthcare IoT threats. By employing DL techniques, Salama et al.^12^ introduce an SDN-enabled outcome in IoT systems. Moreover, the cuda-deep NN (Cu-DNN), BiLSTM, and CuDNNGRU classification models are also implemented for effectual ID. The method also comprises a 10-fold cross-validation procedure for ensuring the objectivity of the outputs. Hizal et al.^13^ introduce an effectual IDS by utilizing DL methods for IoT networks. Furthermore, identifying and reducing potential safety threats in the IoT environment is also enhanced. A two-level IDS utilizing DL techniques encompassing multi- and binary classifiers is constructed to detect DDoS outbreaks. Kumar et al.^14^ present a new DL-based threat-hunting framework (DLTHF) methodology for safeguarding SD-IoT data and detecting outbreaks. An unsupervised feature extracting unit is initially constructed, integrating data perturbation- and normalization-based encoding and scaling in the LSTM Contractive Sparse AE (LSTMCSAE) technique for filtering and transforming values into a standard format. Moreover, a threat detecting system (TDS) employing Multi-Head Self-Attention-assisted BiRNN (MhSaBiGRNN) is introduced for detecting cyber threats and their kinds. Balaji et al.^15^ present a DL technique depending on SqueezeNet and NasNet to detect intrusions in a healthcare setting. SqueezeNet is also used to extract more significant features. Furthermore, network breaches while the NasNet-based classifier detects data transmission across discrete locations. Additionally, the rider optimizer algorithm (ROA) is employed to alter the hyperparameters to detect attacks accurately. Moreover, the auxiliary classifier generative adversarial network (ACGAN) model is combined with the presented model to avoid data imbalance. Alalwany et al.^16^ introduce a novel IDS system for the IoMT environment. The methodology utilizes ML and DL methods, utilizing a stacking fusion methodology to improve ID accuracy by incorporating the merits of various classifiers. The IDS is used inside the Kappa module to ensure real-time accomplishment, facilitating the seamless analysis and immediate response to IoMT data. El.

Shafeiy et al.^17^ propose DCGR_IoT, an IDS system based on the DNN technique for protecting bidirectional transmission networking in the IoT. This technique utilizes advanced methods for improving ID abilities. CNN methodology is used to extract and filter repeated data to enhance effectualness. Moreover, the temporal extracting module implements complex gated recurrent networking (CGRNs). Furthermore, the CGRN methodology is utilized to construct multi-dimensional feature subsets. Heidari, Jamali, and Navimipour^18^ introduce a Colored Timed Petri Nets (CTPN) model for formal verification and performance analysis of the Ad hoc On-Demand Distance Vector (AODV) routing protocol in VANETs, showing its efficiency in real-world scenarios and ensuring efficient routing discovery. Heidari, Navimipour, and Unal^19^ propose a BC-based radial basis function neural networks (RBFNN) model for improving intrusion detection in IoD networks, improving data integrity, and enabling decentralized predictive analytics while outperforming existing methods. Mazid et al.^20^ present the federated learning (FL)-based intrusion detection and privacy preservation (FL-IDPP) methodology for proactive intrusion detection in IoT networks, utilizing FL and bidirectional recurrent neural networks (RNNs) to ensure data privacy and improve accuracy in anomaly detection. Mazid, Kirmani, and Abid^21^ propose a novel approach integrating principal component analysis (PCA), Pearson correlation coefficient (PCC), and convolutional neural networks (CNN) for improving security in IoT networks by mitigating data dimensionality, choosing correlated features, and enhancing the performance of network IDS. Manh et al.^22^ introduce a privacy-preserving cyberattack detection framework for blockchain-based IoT systems, utilizing AI-driven detection modules, homomorphic encryption, single-instruction-multiple-data (SIMD) packing, and FL for effectual, privacy-preserving real-time attack detection. Ferrag et al.^23^ present SecurityBERT, a novel architecture using the Bidirectional Encoder Representations from Transformers (BERT) model and Privacy-Preserving Fixed-Length Encoding (PPFLE) technique for improved cyberthreat detection in IoT networks. Sorour et al.^24^ propose a decentralized framework for IoT intrusion detection, utilizing the joint strategy optimization (JSO) approach for hyperparameter optimization, long short-term memory (LSTM)-based architecture for pattern recognition, and FL for privacy-preserving collaborative training, ensuring robust security against growing cyberthreats. Chen and Sathiyanarayanan^25^ introduce RBoost-Net, a hybrid DL method incorporating ResNet for feature extraction and LightGBM for classification, attaining superior performance in cybersecurity threat detection in IoT environments. Ahmad et al.^26^ introduce VECGLSTM, an advanced attack detection model incorporating variable LSTM (VLSTM), capsule networks, and enhanced gannet optimization algorithm (EGOA), with chaotic cryptographic pelican tunicate swarm optimization (CCPTSO) model for privacy-preserving key management. Bergies et al.^27^ propose a novel IoT architecture integrating model predictive control and deep neural networks (DNNs) to improve performance, ensuring timely threat detection and data privacy through homomorphic encryption.

Bakro et al.^28^ developed an improved cloud IDS by addressing imbalanced data using the synthetic minority over-sampling technique (SMOTE) and applying a hybrid feature selection approach that integrates information gain (IG), chi-square (CS), and particle swarm optimization (PSO) for effectual attack detection. The random forest (RF) model is used for classification, achieving high accuracy in multi-class scenarios. Saheed and Chukwuere^29^ developed a privacy-preserving IDS for cyber-physical industrial IoT (CPS-IIoT) using the pearson correlation coefficient, agglomerative clustering, and a Bidirectional LSTM (BiLSTM) network integrated with a scaled dot-product attention mechanism (AM). Bakro et al.^30^ proposed a hybrid feature selection approach incorporating the grasshopper optimization algorithm (GOA) and genetic algorithm (GA), along with RF classifier. The approach addresses imbalanced data using ADASYN and RUS, attaining high accuracy in multi-class classification across multiple datasets. Abdulganiyu et al.^31^ developed the class-wise focal loss, variational autoencoder, and extreme gradient boosting-based model (CWFLAM-VAE), an attention-driven multi-model architecture. The model addresses the class imbalance issue by generating synthetic rare-class attack data while preserving the original feature distribution. Bakro et al.^32^ developed a cloud IDS that improves classification accuracy by improving feature selection and optimizing an ensemble model using the crow search algorithm (CSA), evaluated on multiple datasets for robust performance across diverse metrics. Kumar et al.^33^ proposed a ML-based hybrid privacy-enhancing technique for detecting attacks against confidentiality in edge computing (PETDA2C-EC), designed to detect attacks targeting the confidentiality of data in Edge-Internet of Things (IoT) environments. Saheed and Misra^34^ developed an IDS system for IoT networks using a gray wolf optimizer (GWO)-based ensemble learning (EL) model that integrates feature selection and extraction, employs a voting mechanism for classification. Saheed, Omole, and Sabit^35^ developed a GA–Modified Adaptive Moment Estimation–Industrial IoT (GA-mADAM-IIoT) model integrating GA for feature selection, a mADAM model for training LSTM networks, and an AM model to improve threat detection. Saheed, Abdulganiyu, and Ait Tchakoucht^36^ developed IoT-Defender, a lightweight IDS system for IoT networks using a modified GA technique for feature selection and a fine-tuned LSTM model within an edge computing environment. Pimple and Sharma^37^ improved the security of CPS by integrating blockchain, multi-attribute feature selection (MAFS), and EL. Saheed and Chukwuere^38^ developed an explainable ensemble transfer learning model. The method integrates Shapley Additive Explanations (SHAP) for interpretability, a hybrid BiLSTM with Autoencoders (BiLAE) for dimensionality reduction, and the barnacle mating optimizer (BMO) for hyperparameter tuning of DL models such as residual neural network (ResNet), Inception, Inception ResNet, and MobileNet CNN–transfer learning (CNN-TL), ensuring high detection accuracy and real-time performance. Saheed, Misra, and Chockalingam^39^ proposeed an IDS for industrial control systems (ICS) using an AE for feature dimensionality reduction, trained on network flow data via a deep CNN (DCNN) and LSTM.

Many existing models depend on static and predefined datasets that do not fully capture the growing behaviour of cyber threats, mitigating their ability to generalize in real-world, dynamic environments. Additionally, existing studies mostly face difficulty with scalability, as they fail to effectually handle the growing volume and complexity of data generated by massive IoT networks. Furthermore, while models such as feature selection and EL are utilized, there is still a gap in developing lightweight models that can work efficiently in resource-constrained environments, such as edge computing. Another significant research gap is in enhancing the robustness of IDS against advanced and novel attack strategies, such as those utilizing AI-based evasion methods. Moreover, existing models tend to lack interpretability, making it difficult for system operators to comprehend decision-making processes. Improving model transparency and explainability remains a key research gap to ensure trust and usability in critical applications. Finally, privacy-preserving techniques in IDS are still underdeveloped, and the integration of privacy mechanisms such as homomorphic encryption and FL with IDS models needs additional exploration to secure sensitive data effectively.

## The proposed method

**Fig. 2:**
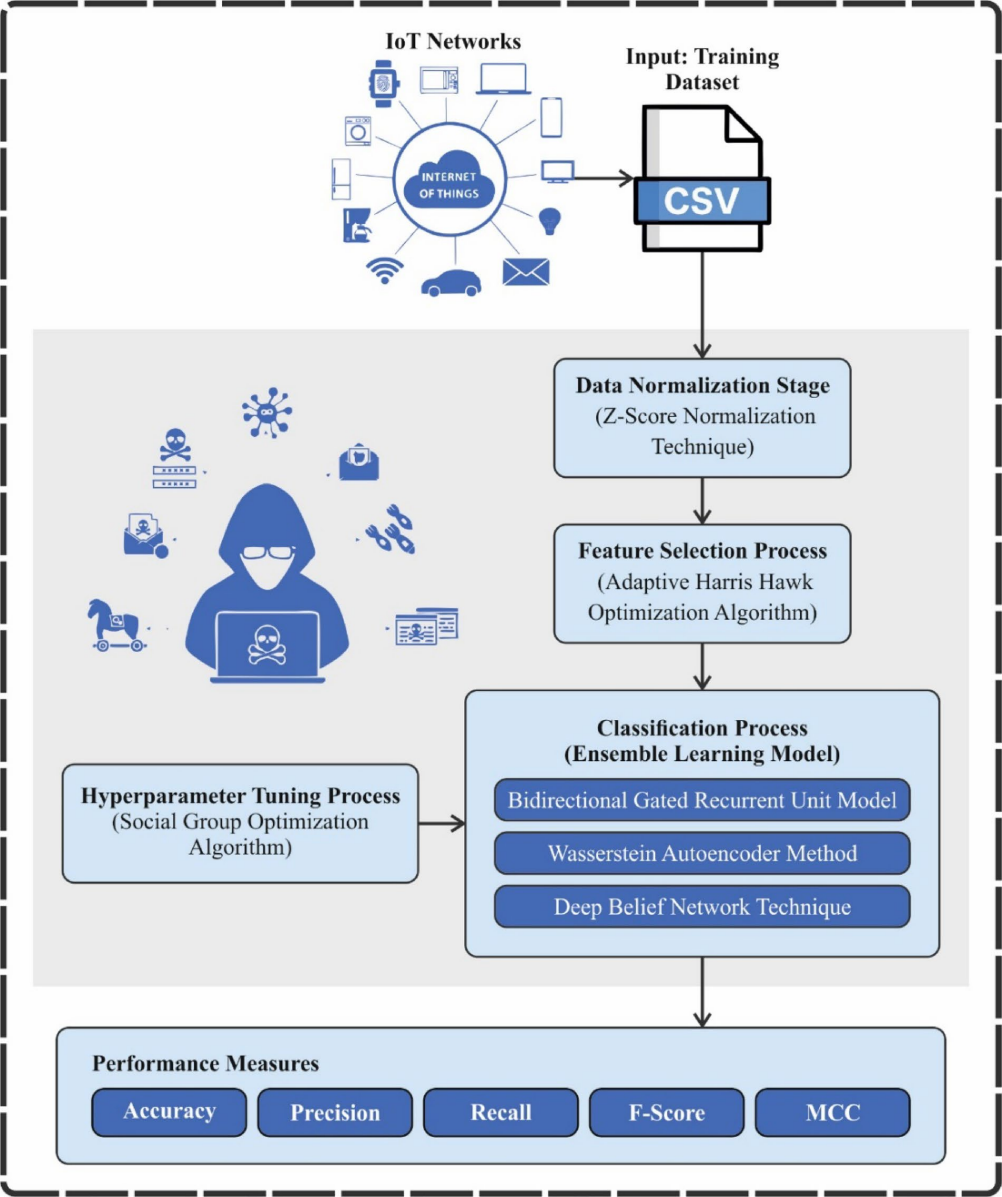
Overall process of AMFS-ELPPCD technique.

This manuscript describes the AMFS-ELPPCD technique, which aims to detect and classify anomalous behaviour for cybersecurity attacks in IoT networks. The method comprises data normalization, feature subset selection, three ensemble classifications, and parameter tuning. Figure 2 represents the overall process of the AMFS-ELPPCD technique.

### Data normalization: Z-score normalization

The data normalization stage initially applies Z-score normalization for converting input data into a beneficial format^40^. This is chosen for its efficiency in transforming data with a mean of zero and a standard deviation of one, standardizing feature scales and enhancing the model’s convergence. Unlike min-max scaling, Z-score normalization is less sensitive to outliers and better handles data with varying discrepancies. The model also confirms that features contribute equally during learning, which is particularly crucial in ensemble DL methods. This technique improves training stability and accelerates gradient-based optimization. It is specifically beneficial in heterogeneous IoT datasets where sensor readings vary widely. Overall, it gives a robust foundation for consistent and reliable classification performance.

This paper used a dataset that includes different features associated with attacks. Numerous pre-processing methods were applied to guarantee the data is appropriate for modelling and analysis, like the reflection of missing values with zeros and the removal of rows. The label encoder or one-hot encoder methods were utilized to convert categorical data into a numeric form suitable for ML, leading to a machine‐readable dataset. These models were essential to guaranteeing interoperability with dissimilar DL methods. Additionally, scaling and FS were important data preparation models that helped to create an effective methodology. Different DL methods apply Z-score normalization to scaling data packets earlier than input. Utilizing this process, the features are rescaled to have standard features with a mean of 0 and a standard deviation (SD) of 1. Utilizing the scikit‐learn average scaler module, feature scaling was attained by transforming autonomous modules into the distribution with standard deviation $\:\left(\sigma\:\right)$ and mean $\:\left(\mu\:\right)$ as presented in Eqs. (1) and (2):

1 $$\:\mu\:=\frac{1}{N}{\sum\:}_{1=1}^{N}({x}_{i})$$

2 $$\:\sigma\:=\sqrt{\frac{1}{N}{\sum\:}_{i=1}^{N}({x}_{i}-\mu\:{)}^{2}})$$

The mean $\:\mu\:$ is computed in Eq. (1), where $\:{x}_{i}$ represents each data value and $\:N$ is the total number of data points; it also provides the average dataset value. The standard deviation $\:\sigma\:$ is evaluated in Eq. (2), where $\:\mu\:$ is the mean and $\:{x}_{i}$ is each individual value; it quantifies how much the data deviates from the mean.

### Feature subset selection: AHHO technique

**Fig. 3:**
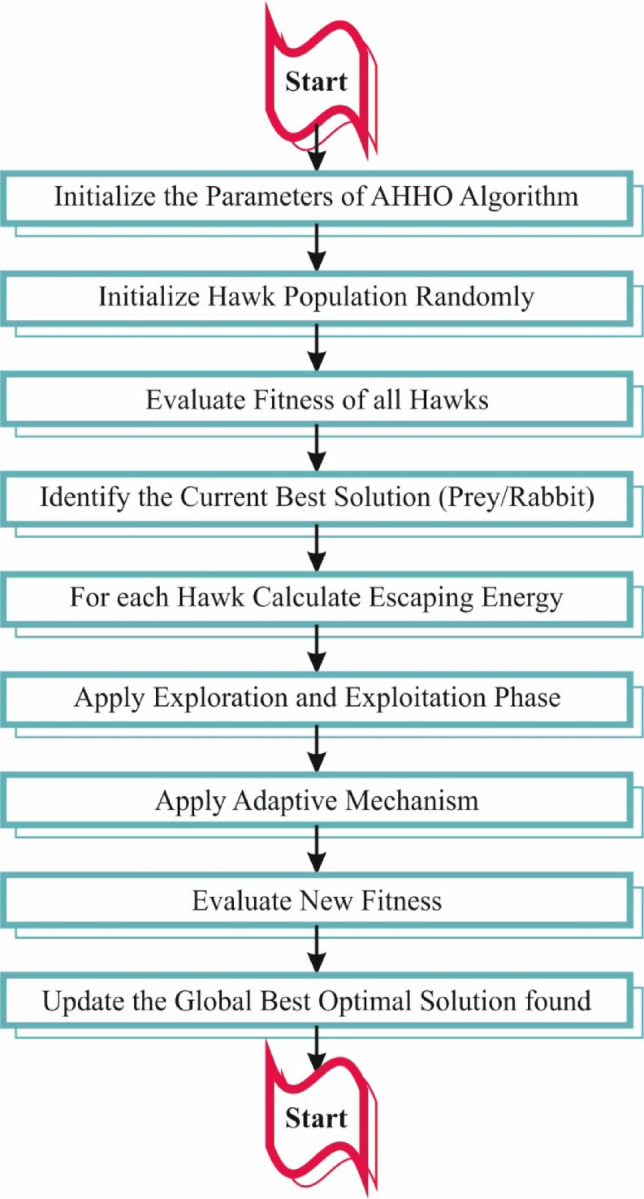
Overall flow of the AHHO technique.

For the subset selection of the feature process, the proposed AMFS-ELPPCD model designs AHHO^41^. This model is chosen because it can effectively balance exploration and exploitation during the search process. AHHO is an advanced optimization algorithm that adapts its strategy based on the complexity of the problem, making it appropriate for high-dimensional datasets. It also assists in reducing the feature space by choosing the most relevant features and enhancing the model’s performance by eliminating redundant or irrelevant features. The adaptability of the AHHO model improves convergence speed and averts local optima, making it superior to conventional methods like GAs or PSO. It also gives robustness in handling noisy data, which is significant for real-world IoT applications. Therefore, AHHO confirms more efficient training and improved generalization across diverse attack scenarios. Figure 3 depicts the flow of the AHHO methodology.

In contrast with Harris Hawk’s searching method, which is frequently identified as “the unexpected pounce” or “the seven kills,” the AHHO metaheuristic approach was designed. There are two critical stages in the HHO modelling procedure (exploration and exploitation). The initial theory establishes that Harris Hawks would tackle living anywhere within a short range of group families. Prey will perform as features, and hawks as hunters. Based on the fitness function (FF), the best features are selected to utilize the AHHO model. The equation to compute an FF is:

3 $$\:fitness\:function=\alpha\:*\zeta\:+(1-\alpha\:)*\frac{/{M}_{e}/}{/{M}_{d}/}$$

The error value represented in the classification is shown in Eq. (3). $\:Me$ is the total number of features removed from the feature extraction phase, $\:Me$ within the presented dataset and specifies the parameter influencing the result of the classification stage. $\:\alpha\:$ represents the weight coefficient and $\:\zeta\:$ is the performance metric. Equation (4) signifies this state below.

4 $$\:Y\left(s+1\right)=\left\{\begin{array}{l}{Y}_{ran}\left(s\right)-{r}_{1}\bullet\:\left|{Y}_{ran}\left(s\right)-2{r}_{2}\bullet\:Y\left(s\right)\right|,\:if\:q\ge\:0.5\\\:\left({Y}_{features}\left(s\right)-{Y}_{n}\right)-{r}_{3}\bullet\:\left(LV+{r}_{4}\bullet\:UV\right),\:if\:q\ge\:0.5\end{array}\right.$$

The location vector of the search agent, $\:Y(s+1)$, is characterized by $\:Y\left(s\right)$, the recent search agent’s location vector, $\:{Y}_{ran}$(s), a randomly selected search agent from the present population, and $\:{r}_{1},$ $\:{r}_{2},$ $\:{r}_{3},$ $\:{r}_{4}$, and $\:p$ denotes random numbers of $\:\left(\text{0,1}\right)$. The location of the features is specified by $\:{y}_{features}\left(s\right)$, while $\:UB$ and $\:LV$ refer to lower and upper variables. The average position of the search agent population now is $\:{Y}_{n}$.

It is recommended that the search agent goes back to the location of a feature or $\:{y}_{features}$ when it crosses the allowable limits, which is the best course of action as demonstrated:

5 $$\:Y\left(s+1\right)\left\{\begin{array}{l}Y\left(s+1\right),\:{if\:Y}_{\text{m}\text{i}\text{n}}\le\:Y\left(s+1\right)\le\:{Y}_{\text{m}\text{a}\text{x}}\\\:{Y}_{features}\left(s\right),\:Y\left(s+1\right)<{Y}_{\text{m}\text{i}\text{n}}\:\\\:{Y}_{features}\left(s\right),\:Y\left(s+1\right)>{Y}_{\text{m}\text{a}\text{x}}\end{array}\right.$$

An AHHO-based feature selection model improves the choice of features, thus decreasing the computational efficiency of the classification procedure.

### Classification process: three ensemble models

Besides, ensemble models such as the BiGRU model, WAE method, and DBN technique are used for the classification process. The ensemble model was chosen due to its capacity to utilize the merits of diverse model types, improving overall performance. The BiGRU model outperforms capturing sequential patterns and temporal dependencies, making it ideal for time-series data in cyberattack detection. WAE assists in capturing complex, non-linear data dispersions through its robust feature learning capabilities, while DBN contributes by learning hierarchical representations and modelling deep features. Integrating these models allows the system to understand temporal and non-temporal patterns, resulting in more accurate and reliable classification outputs. This ensemble approach overcomes the limitations of individual models, enhancing robustness and adaptability to diverse attack patterns. The synergy between these techniques also confirms enhanced generalization, making the model highly efficient in dynamic and complex environments.

#### BiGRU model

With its reset gate model and different update gates, GRU understands the memory functions and forgetting for long-range possessions of input data^42^. This model comprises parameters such as the number of hidden units, learning rate, batch size, dropout rate, and the number of layers. These parameters are key to controlling the capability of the model in capturing sequential patterns and prevent overfitting. Contrary to RNNs and LSTM models, it can resolve the gradient explosion task, contains an added direct architecture, and is quicker and more suitable for learning. The GRU model’s inputs are the feature vector $\:{x}_{t}$ and hidden layer (HL) $\:{h}_{f}$ from the prior time step, which is demonstrated here. The computation process is stated as shown:

6 $$\:{u}_{t}=sigmoid\left({W}_{u}\cdot\:\left[{h}_{t-1},{x}_{t}\right]\right)$$

7 $$\:{r}_{t}=sigmoid\left({W}_{r}\cdot\:\left[{h}_{t-1},{x}_{t}\right]\right)$$

8 $$\:\stackrel{\sim}{{h}_{t}}=\text{t}\text{a}\text{n}\text{h}\left({W}_{h}\cdot\:\left[{r}_{t}\odot\:{h}_{t-1},{x}_{t}\right]\right)$$

9 $$\:h={u}_{t}\odot\:\stackrel{\sim}{{h}_{t}}+\left(1-{u}_{t}\right)\odot\:{h}_{t-1}$$

**Fig. 4:**
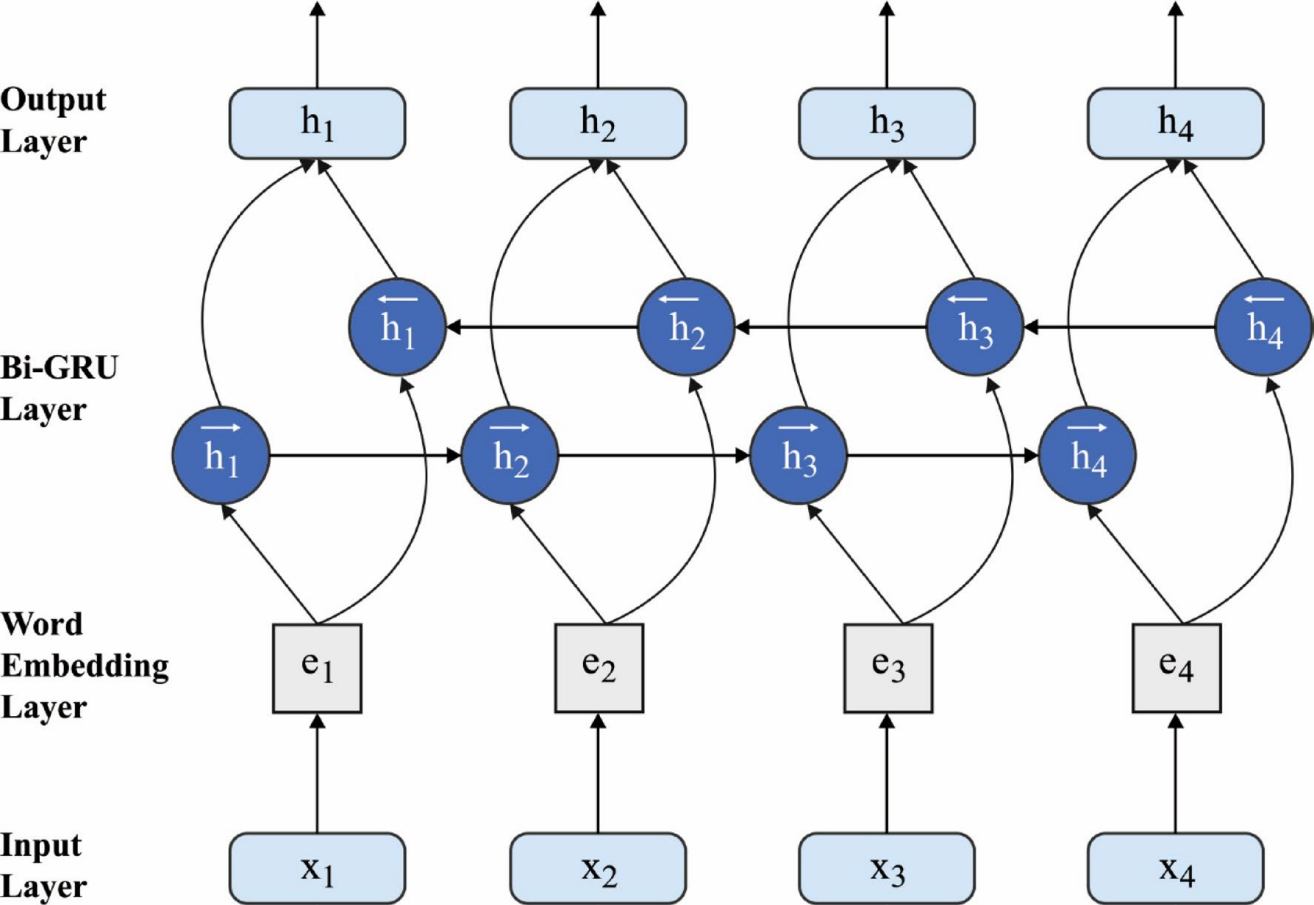
BiGRU architecture.

Meanwhile, $\:{W}_{u},$ $\:{W}_{r}$, and $\:{W}_{h}$ represent weighted matrices of the GRU. Bi-GRU creates a bi-directional RNN architecture by stacking dual Bi-GRU layers and processing the series of inputs in backward and forward orders. It deliberates the present instant data and the data after the present instant. It contains robust memory capabilities and enhanced procedures for temporary time series signals with longer correspondence features. Equations (6)-(9) is applied to calculate the novel HL, $\:\stackrel{\sim}{{h}_{t}}$. Considering that a similar group of parameters is applied to calculate every time step, a unique collection of parameters, $\:{W}_{u},$ $\:{W}_{r}$, and $\:{W}_{h}$, should be enhanced. This decreases the complexity training of the GRU since the parameter count is separated from time steps. Figure 4 shows the infrastructure of BiGRU.

#### WAE model

Like VAE, WAE designs for minimizing either the regularization term or the reconstruction cost, signified as $\:{D}_{z}({P}_{z},\:{Q}_{z})$, demonstrating some random divergence metrics amongst $\:{Q}_{z}$ and $\:{P}_{z}$^43^. The generic model of the loss function is stated as:

10 $$\:{\mathcal{L}}_{WAE}={\mathbb{E}}_{q\left(z|x\right)}[\text{log}p\left(x|z\right)]+\lambda\:\cdot\:{D}_{z}\left(q\left(z|x\right),p\left(z\right)\right)$$

Whereas $\:{\mathbb{E}}_{q\left(z|x\right)}[\text{log}p\left(x|z\right)]$ acts as the reconstruction term, ${D}_{z}\left(\left(z|x\right)\!,\left(z\right)\right)$ indicates some random divergence metrics, and $\lambda$ > 0 means hyperparameter. The dual particular regularizers, the adversarial training and the Maximum Mean Discrepancy (MMD) are presented to deal with problems and offer greater resilience in latent area modelling, and that is notable that after adverse training is applied, it is associated with the Adversarial AE presented in this section. An Alternative approach is presented to utilize the Sliced-Wasserstein distance to convert the hidden dispersal of the SE into a previous delivery, which might be tested. After this new method, current methods have spotted considerable progressions, as demonstrated. The WAE utilizes the Wasserstein distance to determine straightforward and determinist maps from input data to hidden areas, providing the performances to the essential randomness of hidden variables in either VAEs or GANs.

#### DBN model

The DBN comprises the BPNN layer and some restricted Boltzmann machine (RBM) layers^44^. There are dual-layer RNNs for all RBMs comprising a visible layer (VL) and a HL. The HL neurons are implicitly fully connected (FC) with the VL’s help by weighted values; however, the neurons in the similar layer are not linked. In RBM, all neurons are associated with the state variables. The $\:jth$ hidden and $\:ith$ visible variable is expressed as $\:{h}_{j}$ and$\:\:{v}_{i}$. The RBM parameters are $\:=({a}_{i},{b}_{j},{w}_{ij})$, and $\:{a}_{i}$ and $\:{b}_{j}$ represent the bias of the $\:ith$ and $\:jth$ neurons in the VL and HL, correspondingly; $\:{w}_{ij}$ represents weighting among the $\:ith\:$and$\:\:jth\:$neurons in the VL and HL. For the specific state $\:(v,h)$, the joint coordination energy function amongst the VL and HL is outlined as demonstrated in Eq. (11).

11 $$\:E\left(v,h|\theta\:\right)=-{\sum\:}_{i=1}^{n}{a}_{i}{v}_{i}-{\sum\:}_{j=1}^{m}{b}_{j}{h}_{j}-{\sum\:}_{i=1j}^{n}{\sum\:}_{=1}^{m}{v}_{i}{w}_{ij}{h}_{j}$$

The joint dispersal of some a collection of $\:(v,h)$ fulfils Eq. (12).

12 $$\:P\left(v,h|\theta\:\right)=\frac{\text{e}\text{x}\text{p}\left(-E\left(v,h|\theta\:\right)\right)}{{\varSigma\:}_{v,h}\text{exp}\left(-E\left(v,h|\theta\:\right)\right)}$$

The contrast divergence (CD) model is accepted for pretraining, and the parameters $\:\theta\:$ are continuously upgraded. The DBN training procedure contains dual phases. As explained above, the unsupervised pretraining procedure for all RBMs is the initial phase’s model training of the DBN. After each RMB is wholly trained, a BP classifier layer, in addition to the top layer, is used to output the classification outcomes. Each parameter is fine-tuned and supervised optimally during this second phase by reducing the target loss function.

### Hyperparameter selection: SGO technique

**Fig. 5:**
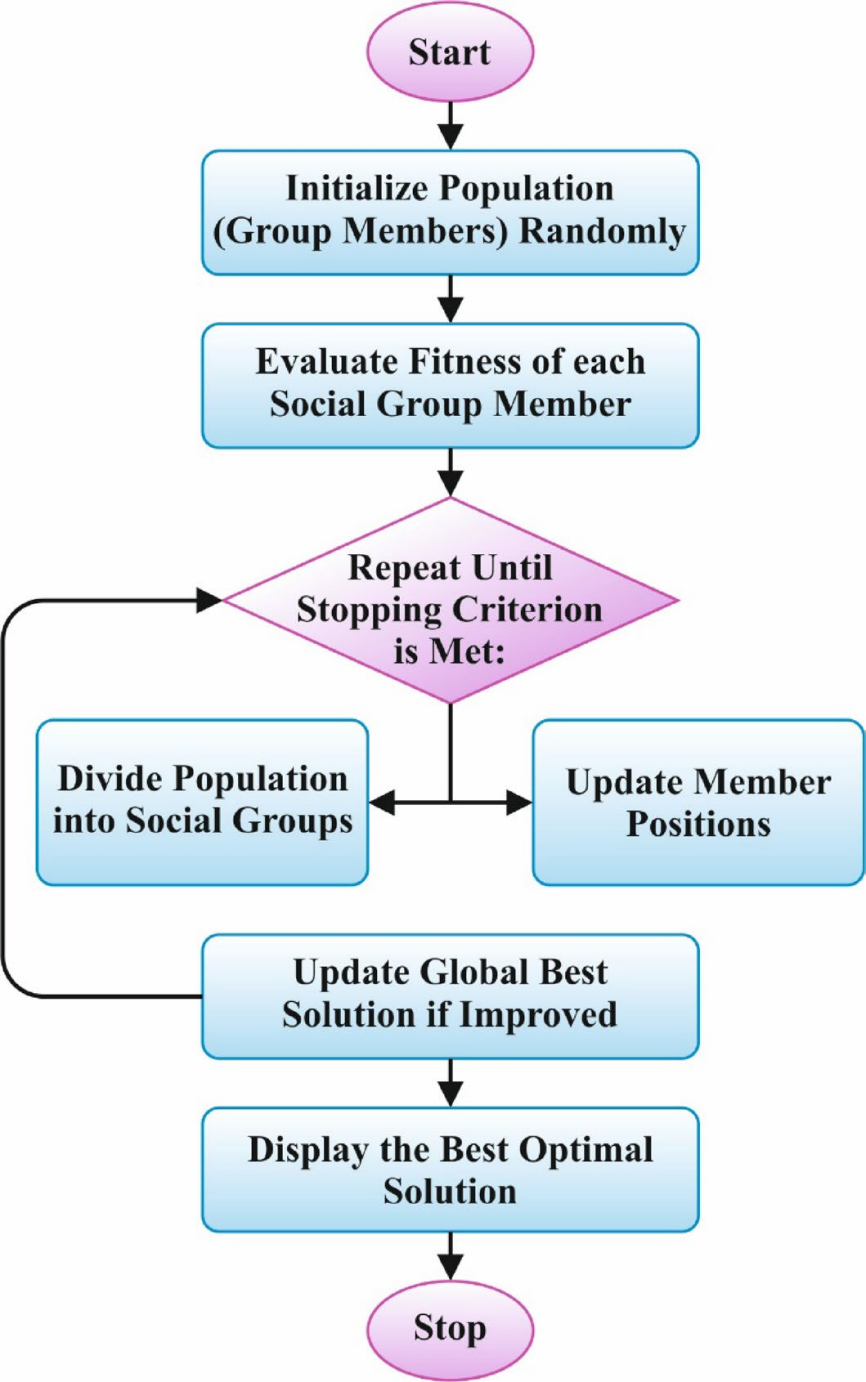
SGO flowchart.

Finally, the SGO optimally adjusts the ensemble classifiers’ hyperparameter values, resulting in more excellent classification performance^45^. This technique is chosen because it can effectively explore and exploit the search space, replicating social behaviours for optimal solutions. Unlike conventional optimization algorithms, SGO employs social interactions to guide the search process, improving the convergence towards optimal hyperparameters. Its flexibility in handling complex, multi-dimensional problems makes it ideal for tuning DL models with multiple parameters. The adaptive nature of the SGO model allows it to fine-tune hyperparameters based on growing data patterns, enhancing the accuracy and adaptability of the technique. Compared to gradient-based optimization methods, SGO is less prone to getting stuck in local minima, resulting in improved generalization and robustness. Furthermore, SGO requires fewer objective function evaluations, making it computationally effective for resource-constrained environments. Figure 5 specifies the flowchart of the SGO model.

SGO is a meta-heuristic model recently presented to solve composite engineering or optimization problems. This model simulates the individual’s behaviour in the social group trying to resolve real-world problems. Theoretically, this goal is obtained by interactions and the alternate when knowledge between SG members. The mathematical model of this theory includes the succeeding representations: $\:N$ epitomizes the individual counts (the population), and $\:m$ denotes trait counts equivalent to the individual inside the group. Every $\:ith$ individual comparable to the solution $\:{X}_{i}=\left[{x}_{1,i},\:{x}_{2,\:i},\:\dots\:,\:{x}_{j,\:i},\:\dots\:{x}_{m,\:i}\right],$ $\:i=\text{1,2},\dots\:,\:N$ from the population, $\:{x}_{j,i}$ denote module $\:j$ of the solution$\:{\:X}_{i}$, and $\:{f}_{i}\left({X}_{i}\right)$ represent FF for the $\:{X}_{i}\:$solution.

This model advances over three stages: acquiring, initialization, and improving. During this initialization stage, solutions $\:{X}_{j}$ are arbitrarily made inside the forced maximum $\:\left({X}_{\text{m}\text{a}\text{x}}\right)$ and minimum $\:\left({X}_{\text{m}\text{i}\text{n}}\right)$ bounds utilizing the succeeding Eq. (13):

13 $$\:{X}_{i}={X}_{\text{m}\text{i}\text{n}}+{r}_{1}\cdot\:\left({x}_{\text{m}\text{a}\text{x}}-{X}_{\text{m}\text{i}\text{n}}\right),\:i=\text{1,2},\:\dots\:N$$

In this stage of improving, to improve the individual traits $\:i\left({X}_{i}^{new}\right)$, the association among the individual and individual $\:i\left({X}_{i}\right)$ by the superior understanding (considering the best individual, $\:{X}_{Best}$) is shown. Solutions are upgraded by the succeeding Eq. (14):

14 $$\:{X}_{i}^{new}=c\cdot\:{X}_{i}+{r}_{2}\cdot\:\left({X}_{Best}-{X}_{i}\right),\:i=\text{1,2},\:\dots\:N$$

The acquiring stage models the knowledge sharing among the $\:ith$ individual ($\:{X}_{j}$) and the best individual $\:\left({X}_{Best}\right)$, in addition to among individuals and individuals $\:r$ chosen randomly from the group ($\:{X}_{r}$). Here, solutions are upgraded according to the implementation of the present $\:{X}_{i}$ solution in comparison with the arbitrarily picked $\:{X}_{r}\:$solution, utilizing the subsequent Eqs.:

15 $$If~f\left( {X_{i} } \right) < f\left( {X_{i} } \right)~Then,~X_{i}^{{new}} = X_{i} + r_{3} \left( {X_{i} - X_{r} } \right) + r_{4} \left( {X_{{Best}} - X_{i} } \right)$$

16 $$\:Else\:{X}_{i}^{new}={X}_{i}+{r}_{3}({X}_{r}-{X}_{i})+{r}_{4}({X}_{Best}-{X}_{i}),i=\text{1,2},\dots\:,N$$

Meanwhile, $\:{X}_{i}^{new}$ refers to novel solutions gained through Eqs. (14)-(16);$\:\:{X}_{Best}$ denotes the individual with the best understanding (the optimal solution); $\:{X}_{r}$ signifies the solution chosen at random from the recent population;$\:\:c$ represents the self-introspection feature $\:\left(0<c<1\right)$.

The SGO model initiates by making a first population utilizing Eq. (13), accompanied by a recurring cycle, which successively achieves the acquiring and improving stages, while solutions are upgraded utilizing Eqs. (14)- (16). Finally, all iterations are checked whether the pre-defined maximal iteration counts $\:({k}_{\text{m}\text{a}\text{x}}$) are attained. When $\:{k}_{\text{m}\text{a}\text{x}}$ can be influenced, the iterative cycle replicates; otherwise, the model ends, recollecting the best solution $\:\left({X}_{Best}\right)$ and its consistent fitness $\:f\left({X}_{Best}\right)$. Algorithm 1 describes the SGO methodology and Table 1 describes the parameters involved in the SGO model.

**Algorithm 1:**
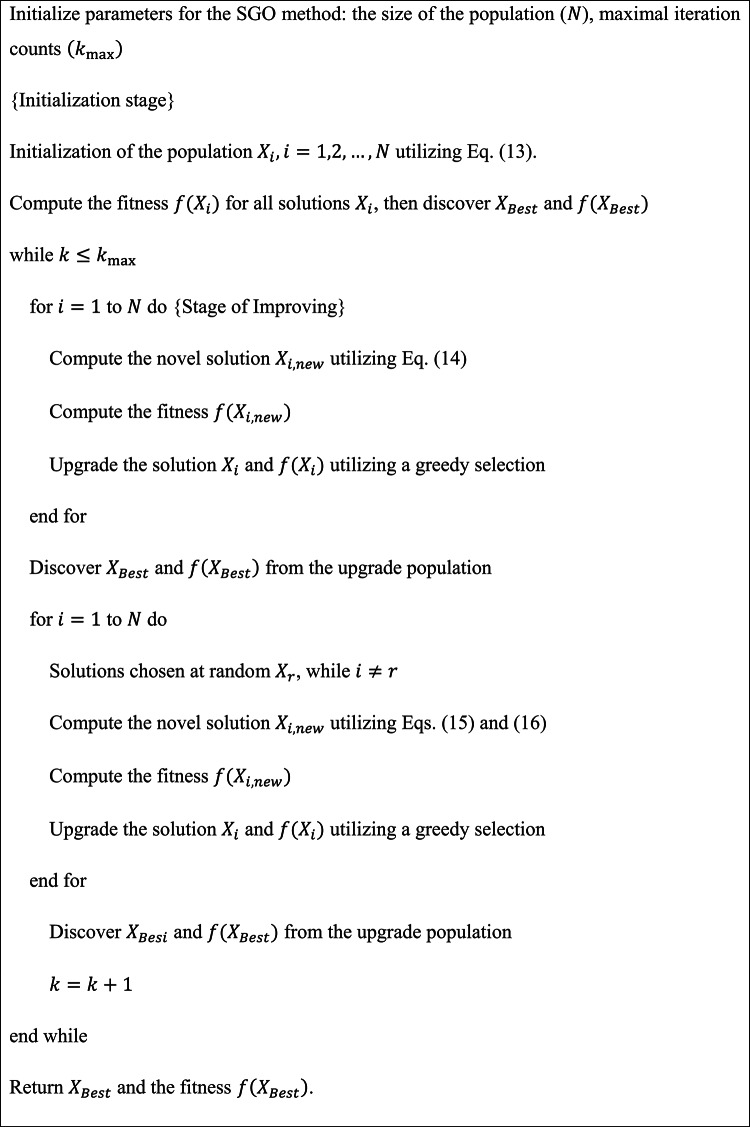
SGO model

**Table 1:** Hyperparameters of the SGO technique.

Hyperparameter	Description	Example Value/Range
POPU_SIZE	Individual numbers or solutions in the populace	30–100
GROUP_NO	Overall social groups in the populace	3–10
MAX_ITER	Maximum iterations for convergence	100–1000
GROUP_INFLUENCE_ FACTOR	Regulates the activities of the group’s best member	0.1–1.0 (e.g., 0.7)
PERSONAL_INFLUENCE_ FACTOR	Controls the self-learning ability of individuals	0.1–1.0 (e.g., 0.3)
SOCIAL_LEARNING_RATE	Learning rate to balance personal and group influence	0.1–0.9
MUTATION_RATE	Probability of presenting arbitrary discrepancies	0.01–0.1
CONVERGENCE_THRESHOLD	Minimum change in fitness to stop iterations early	1e-5–1e-3
OBJECTIVE_FUNCTION	Function used to compute individual fitness	Problem-specific
INIT_STRATEGY	Method utilized to initialize populace	Random uniform or Gaussian

Fitness choice is a substantial factor that influences the performance of the SGO approach. The hyperparameter range procedure contains the solution encrypted system for evaluating the efficiency of the candidate solution. The SGO technique reflects accuracy as the primary measure of projecting the FF.

17 $$\:Fitness\:=\:\text{m}\text{a}\text{x}\:\left(P\right)$$

18 $$\:P=\frac{TP}{TP+FP}$$

Meanwhile, $\:TP$ and $\:FP$ signify the positive values of true and false.

## Experimental result and analysis

The performance validation of the AMFS-ELPPCD method is studied under the CICIDS-2017 dataset^46^. The technique is simulated using the Python 3.6.5 tool on a PC with an i5-8600k, 250GB SSD, GeForce 1050Ti 4GB, 16GB RAM, and 1 TB HDD. The parameter settings are: learning rate: 0.01, activation: ReLU, epoch count: 50, dropout: 0.5, and batch size: 5.

The CICIDS-2017 dataset contains 10,973 records below five class labels, as depicted in Table 2. The total number of features is 78, but only 32 are selected.

**Table 2:** CICIDS-2017 dataset details.

Class Labels	No. of Records
*ICIDS-2017 Dataset*	
Benign	2500
DDoS	2500
DoS	2500
Bot	1966
Web Attack	1507
Total Records	10,973

**Fig. 6:**
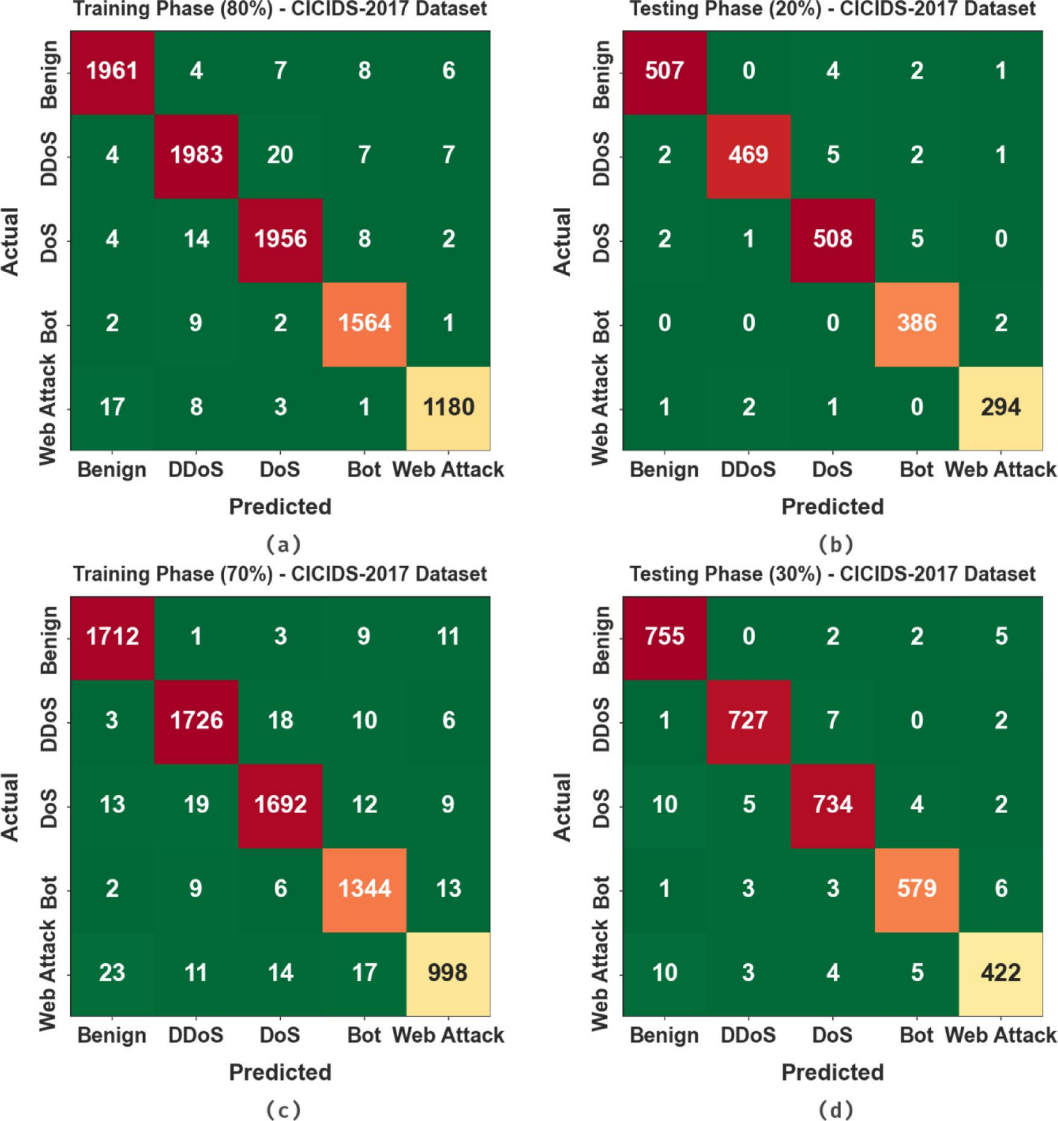
CICIDS-2017 dataset (a-c) 80% and 70%TSPH and (b-d) 20% and 30%TSPH.

Figure 6 presents the confusion matrices for the CICIDS-2017 dataset, highlighting the classification performance of the AMFS-ELPPCD model across diverse attack categories. During the 80% TRPH, the model achieved high accuracy, correctly classifying most instances with few misclassifications in categories such as DoS, DDoS, Web Attack, and Bot. The 70% TRPH also illustrated robust performance, with a slight increase in misclassified samples. In the 20% TSPH, the model exhibited reliable results, accurately detecting both benign and attack instances, including complex types like Web Attack and Bot. Overall, the confusion matrices confirm the robustness and efficiency of the AMFS-ELPPCD method in multi-class cyberattack detection.

**Fig. 7:**
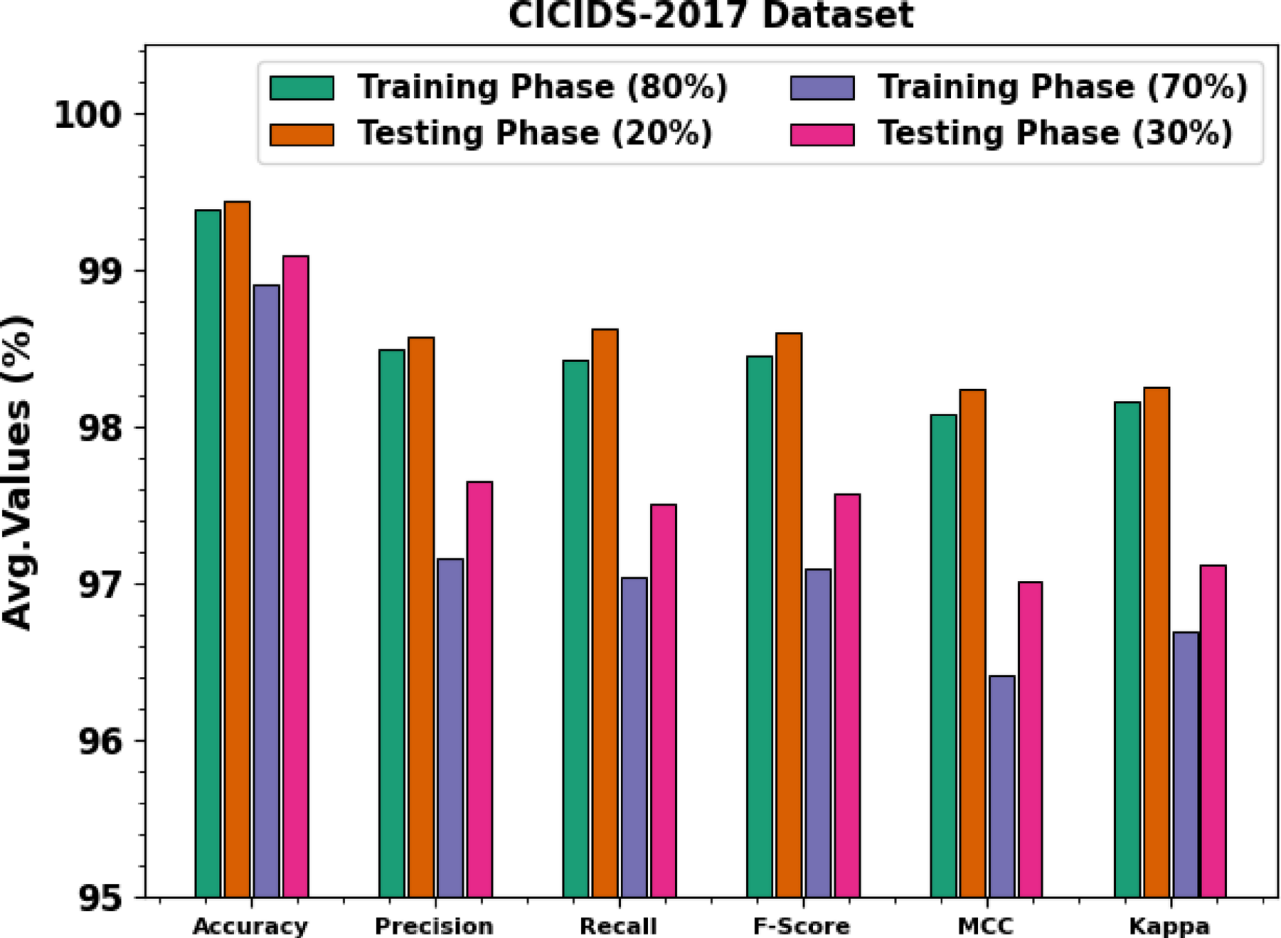
Average of AMFS-ELPPCD model on the CICIDS-2017 dataset.

Table 3; Fig. 7 signify the attack detection of AMFS-ELPPCD methodology on the CICIDS-2017 dataset. The results imply that the AMFS-ELPPCD methodology correctly recognized the samples under 80:20 and 70:30 TRPH/TSPH. With 80%TRPH, the AMFS-ELPPCD technique presents an average $\:acc{u}_{y}$ of 98.49%, $\:pre{c}_{n}$ of 99.39%, $\:rec{a}_{l}$ of 98.43%, $\:{F}_{score}$ 98.46%, $\:MCC\:$of 98.08%, and Kappa of 98.16%, correspondingly. Moreover, with 20%TSPH, the AMFS-ELPPCD technique presents an average $\:acc{u}_{y}$ of 99.44%, $\:pre{c}_{n}$ of 98.57%, $\:rec{a}_{l}$ of 98.63%, $\:{F}_{score}$ of 98.60%, $\:MCC\:$of 98.24%, and Kappa of 98.25%, correspondingly. Besides, with 70%TRPH, the AMFS-ELPPCD technique presents an average $\:acc{u}_{y}$ of 98.91%, $\:pre{c}_{n}$ of 97.16%, $\:rec{a}_{l}$ of 97.04%, $\:{F}_{score}$ of 97.10%, $\:MCC\:$of 96.42%, and Kappa of 96.69% respectively. Also, with 30%TSPH, the AMFS-ELPPCD technique presents an average $\:acc{u}_{y}$ of 99.09%, $\:pre{c}_{n}$ of 97.65%, $\:rec{a}_{l}$ of 97.51%, $\:{F}_{score}$ of 97.58%, $\:MCC\:$of 97.01%, and Kappa of 97.12%, respectively.

**Table 3:** Attack detection of AMFS-ELPPCD model on the CICIDS-2017 dataset.

Class Labels	$\:\varvec{A}\varvec{c}\varvec{c}{\varvec{u}}_{\varvec{y}}$	$\:\varvec{P}\varvec{r}\varvec{e}{\varvec{c}}_{\varvec{n}}$	$\:\varvec{R}\varvec{e}\varvec{c}{\varvec{a}}_{\varvec{l}}$	$\:{\varvec{F}}_{\varvec{s}\varvec{c}\varvec{o}\varvec{r}\varvec{e}}$	$\:\varvec{M}\varvec{C}\varvec{C}$	Kappa
*TRPH (80%)*						
Benign	99.41	98.64	98.74	98.69	98.31	98.05
DDoS	99.17	98.27	98.12	98.19	97.65	98.06
DoS	99.32	98.39	98.59	98.49	98.05	98.61
Bot	99.57	98.49	99.11	98.80	98.54	98.07
Web Attack	99.49	98.66	97.60	98.13	97.83	98.01
Average	99.39	98.49	98.43	98.46	98.08	98.16
*TSPH (20%)*						
Benign	99.45	99.02	98.64	98.83	98.47	98.03
DDoS	99.41	99.36	97.91	98.63	98.26	98.86
DoS	99.18	98.07	98.45	98.26	97.72	98.18
Bot	99.50	97.72	99.48	98.60	98.30	98.06
Web Attack	99.64	98.66	98.66	98.66	98.45	98.13
Average	99.44	98.57	98.63	98.60	98.24	98.25
*TRPH (70%)*						
Benign	99.15	97.66	98.62	98.14	97.59	98.67
DDoS	99.00	97.73	97.90	97.82	97.17	92.72
DoS	98.78	97.63	96.96	97.30	96.51	96.65
Bot	98.98	96.55	97.82	97.18	96.56	98.65
Web Attack	98.65	96.24	93.89	95.05	94.27	96.74
Average	98.91	97.16	97.04	97.10	96.42	96.69
*TSPH (30%)*						
Benign	99.06	97.17	98.82	97.99	97.38	91.77
DDoS	99.36	98.51	98.64	98.58	98.17	98.61
DoS	98.88	97.87	97.22	97.54	96.81	98.80
Bot	99.27	98.14	97.80	97.97	97.53	97.79
Web Attack	98.88	96.57	95.05	95.80	95.16	98.61
Average	99.09	97.65	97.51	97.58	97.01	97.12

**Fig. 8:**
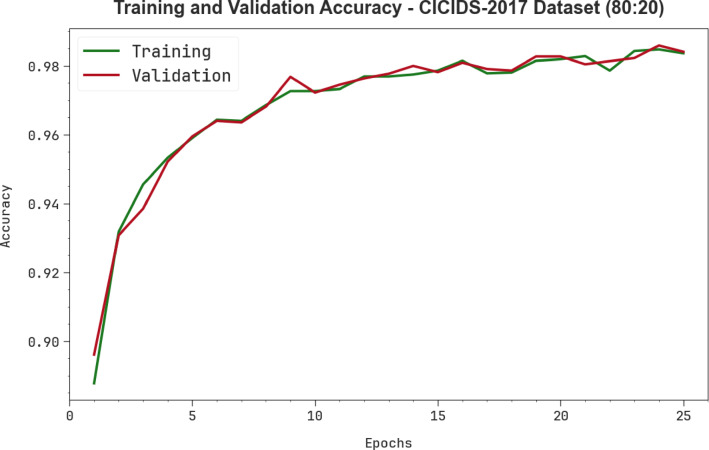
$\:Acc{u}_{y}$ analysis of AMFS-ELPPCD approach on the CICIDS-2017 dataset.

Figure 8 illustrates the training (TRA) $\:acc{u}_{y}$ and validation (VAL) $\:acc{u}_{y}$ results of the AMFS-ELPPCD methodology on the CICIDS-2017 dataset below 80%TRPH and 20%TSPH. The $\:acc{u}_{y}\:$analysis is calculated across a period of 0–25 epochs. The figure highlights that the TRA and VAL $\:acc{u}_{y}$ values exhibit a rising trend, which informs the ability of the AMFS-ELPPCD approach with superior outcomes over several iterations. Simultaneously, the TRA and VAL $\:acc{u}_{y}$ remain closer across the epochs, which directs minimum overfitting and exhibitions maximal outcomes of the AMFS-ELPPCD approach, guaranteeing continuous prediction on hidden samples.

**Fig. 9:**
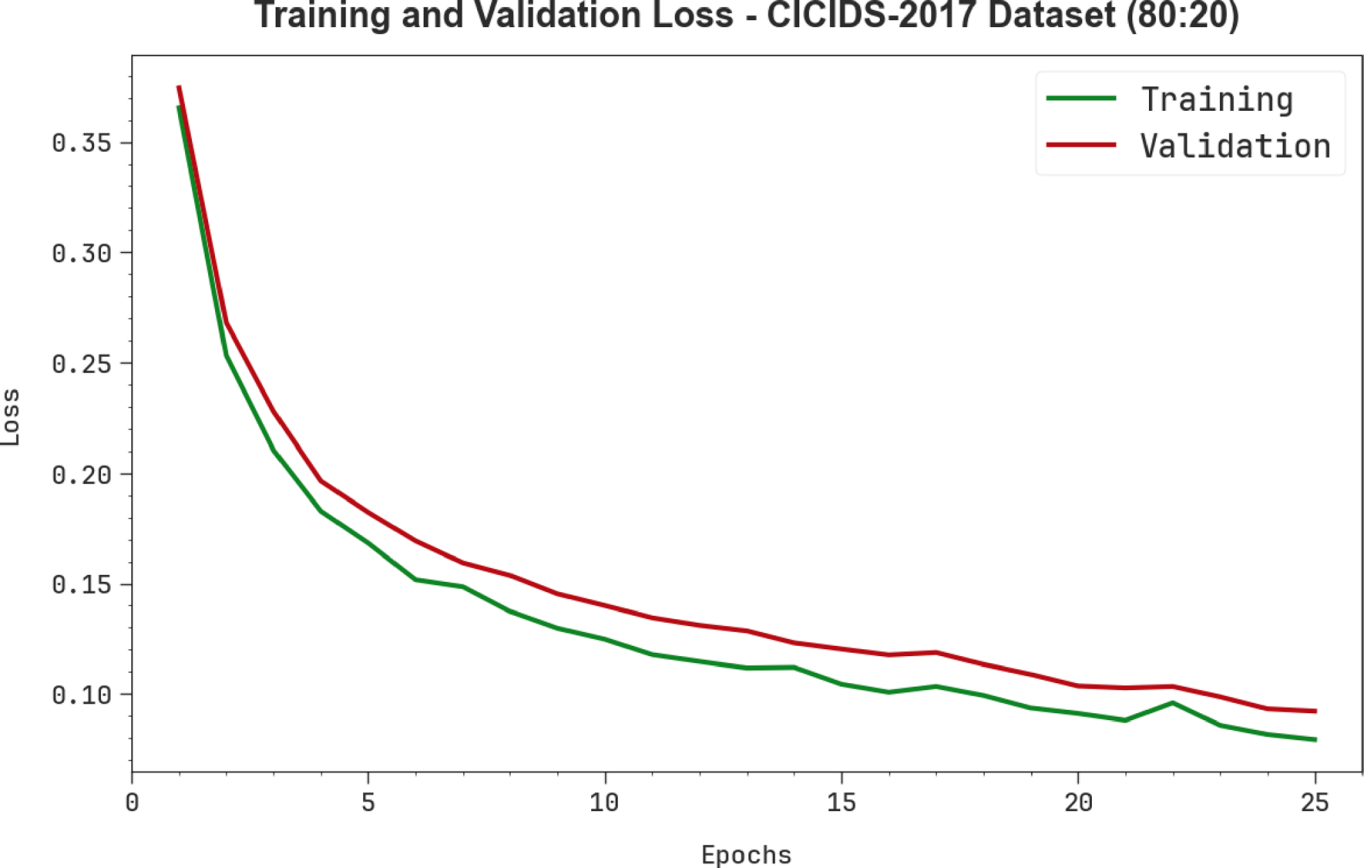
Loss graph of AMFS-ELPPCD model on CICIDS-2017 dataset.

Figure 9 shows the TRA loss (TRALOS) and VAL loss (VALLOS) curves of the AMFS-ELPPCD technique on the CICIDS-2017 dataset below 80%TRPH and 20%TSPH. The loss values are calculated across an interval of 0–25 epochs. The TRALOS and VALLOS analysis illustrates a decreasing trend, informing the capability of the AMFS-ELPPCD method in balancing a trade-off between simplification and data fitting. The continuous reduction in loss values assures the optimal outcomes of the AMFS-ELPPCD method and tuning of the prediction results over time.

**Fig. 10:**
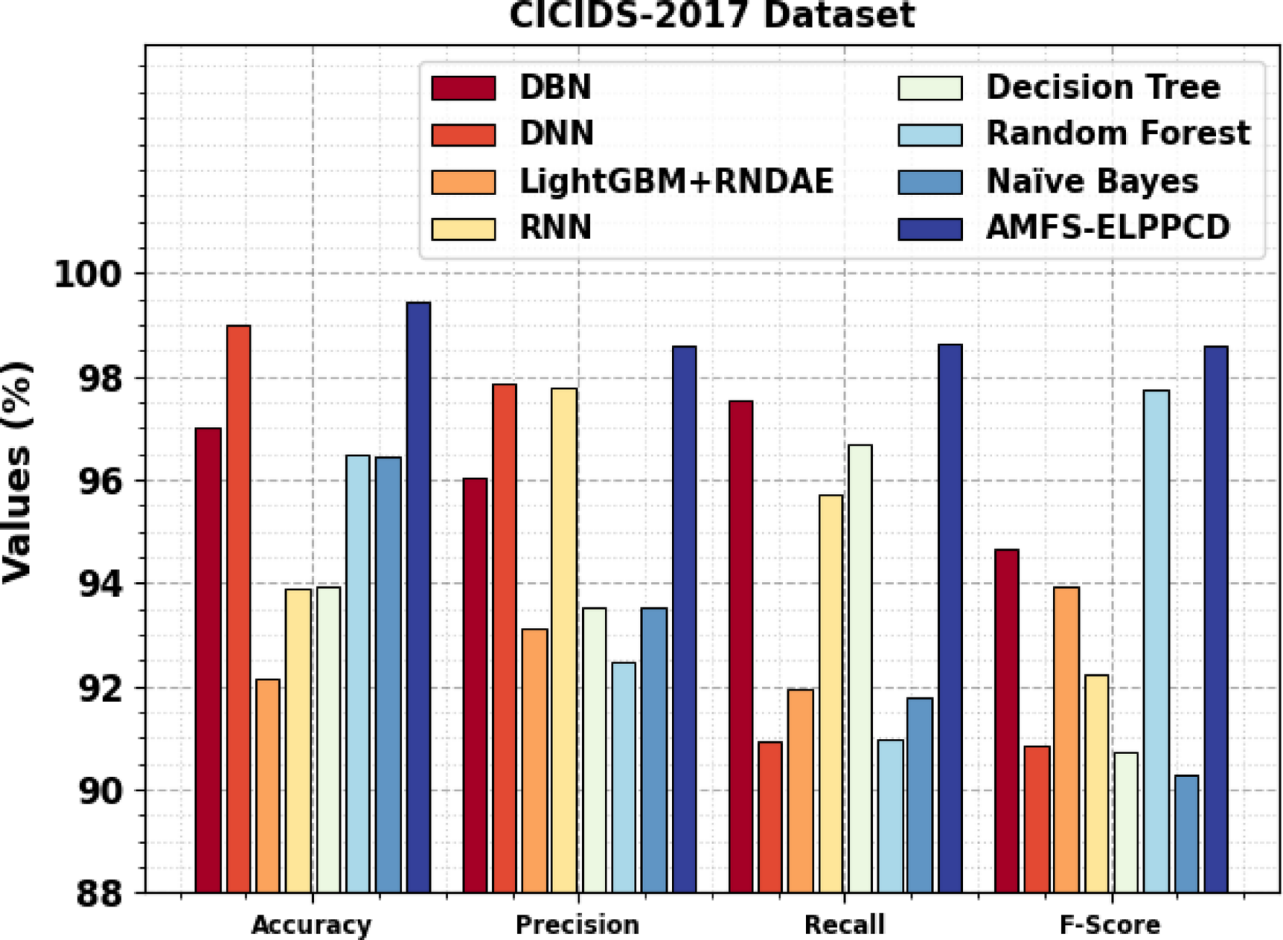
Comparative analysis of AMFS-ELPPCD model on CICIDS-2017dataset.

Table 4; Fig. 10 inspect the comparison study of AMFS-ELPPCD methodology on the CICIDS-2017 dataset with existing approaches. The results highlight that the DBN, DNN, LightGBM + RNDAE, RNN, Decision Tree (DT), RF, and Naïve Bayes (NB) models have reported lower performance. At the same time, the proposed AMFS-ELPPCD method reported maximum performance with a high $\:acc{u}_{y}$ of 99.44%, $\:pre{c}_{n}$ of 98.57%, $\:rec{a}_{l}$ of 98.63%, and $\:{F}_{score}$ of 98.60%, correspondingly.

**Table 4:** Comparative analysis of AMFS-ELPPCD model on CICIDS-2017dataset.

**Model**	$\:\varvec{A}\varvec{c}\varvec{c}{\varvec{u}}_{\varvec{y}}$	$\:\varvec{P}\varvec{r}\varvec{e}{\varvec{c}}_{\varvec{n}}$	$\:\varvec{R}\varvec{e}\varvec{c}{\varvec{a}}_{\varvec{l}}$	$\:{\varvec{F}}_{\varvec{s}\varvec{c}\varvec{o}\varvec{r}\varvec{e}}$
*CICIDS-2017 Dataset*				
DBN	97.00	96.03	97.55	94.65
DNN	98.98	97.86	90.94	90.86
LightGBM + RNDAE	92.14	93.12	91.96	93.94
RNN	93.87	97.76	95.73	92.24
DT	93.93	93.54	96.68	90.71
RF	96.48	92.46	90.95	97.72
NB	96.45	93.51	91.79	90.28
AMFS-ELPPCD	99.44	98.57	98.63	98.60

**Fig. 11:**
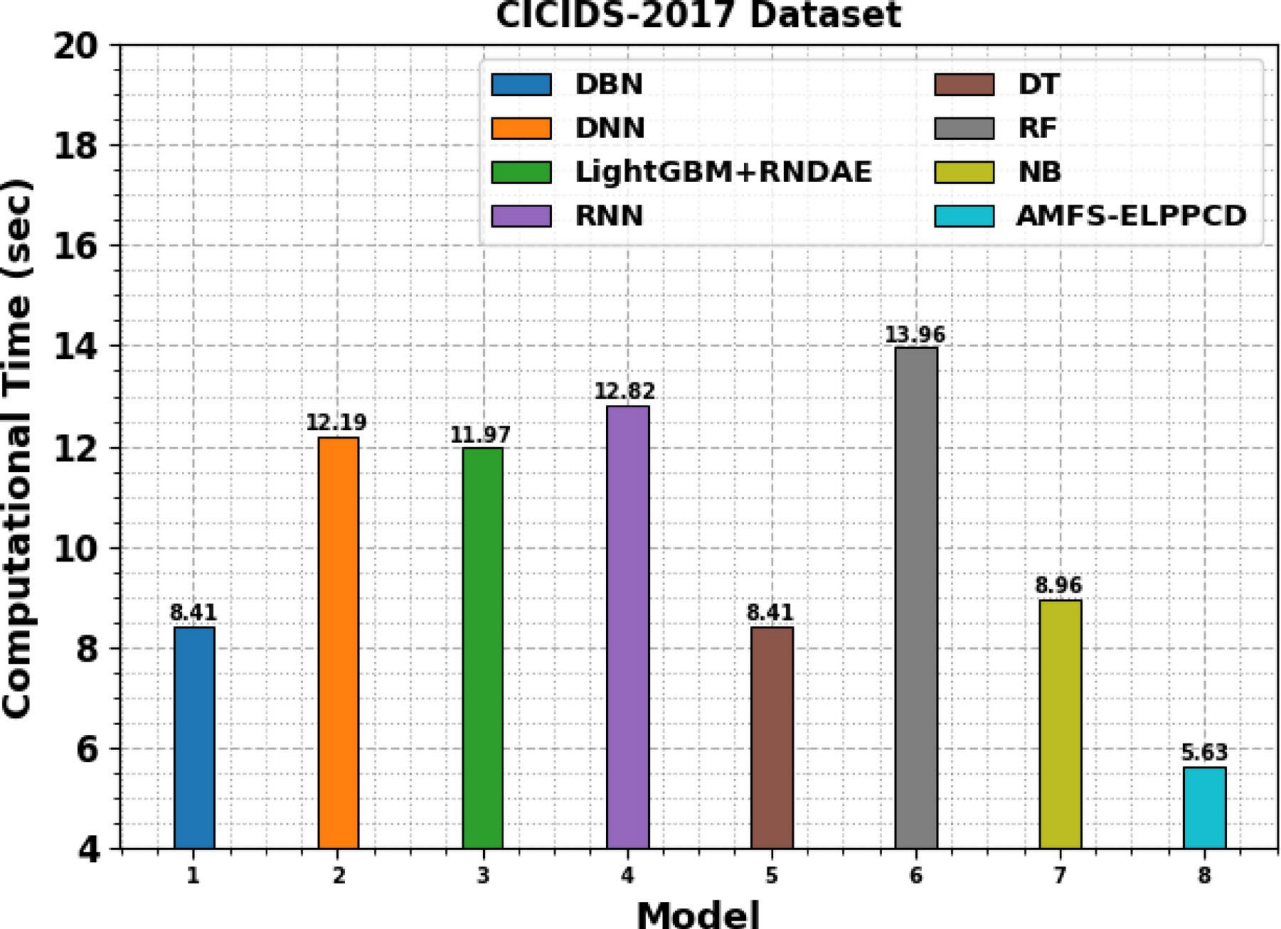
CT analysis of AMFS-ELPPCD method with existing models.

Table 5; Fig. 11 illustrates the computational time (CT) analysis of the AMFS-ELPPCD method with existing models. The AMFS-ELPPCD method demonstrated the lowest CT of 5.63 s, indicating superior efficiency. Conventional models such as DT and DBN recorded a CT of 8.41 s, while NB followed closely with 8.96 s. DNN and LightGBM combined with RNDAE illustrated higher CTs of 12.19 and 11.97 s respectively. RNN exhibited the highest CT at 12.82 s, and RF required 13.96 s. These results highlight the computational advantage of the AMFS-ELPPCD model over existing methods.

**Table 5:** CT analysis of AMFS-ELPPCD method with existing models.

Model	CT (sec)
*CICIDS-2017 Dataset*	
DBN	8.41
DNN	12.19
LightGBM + RNDAE	11.97
RNN	12.82
DT	8.41
RF	13.96
NB	8.96
AMFS-ELPPCD	5.63

**Fig. 12:**
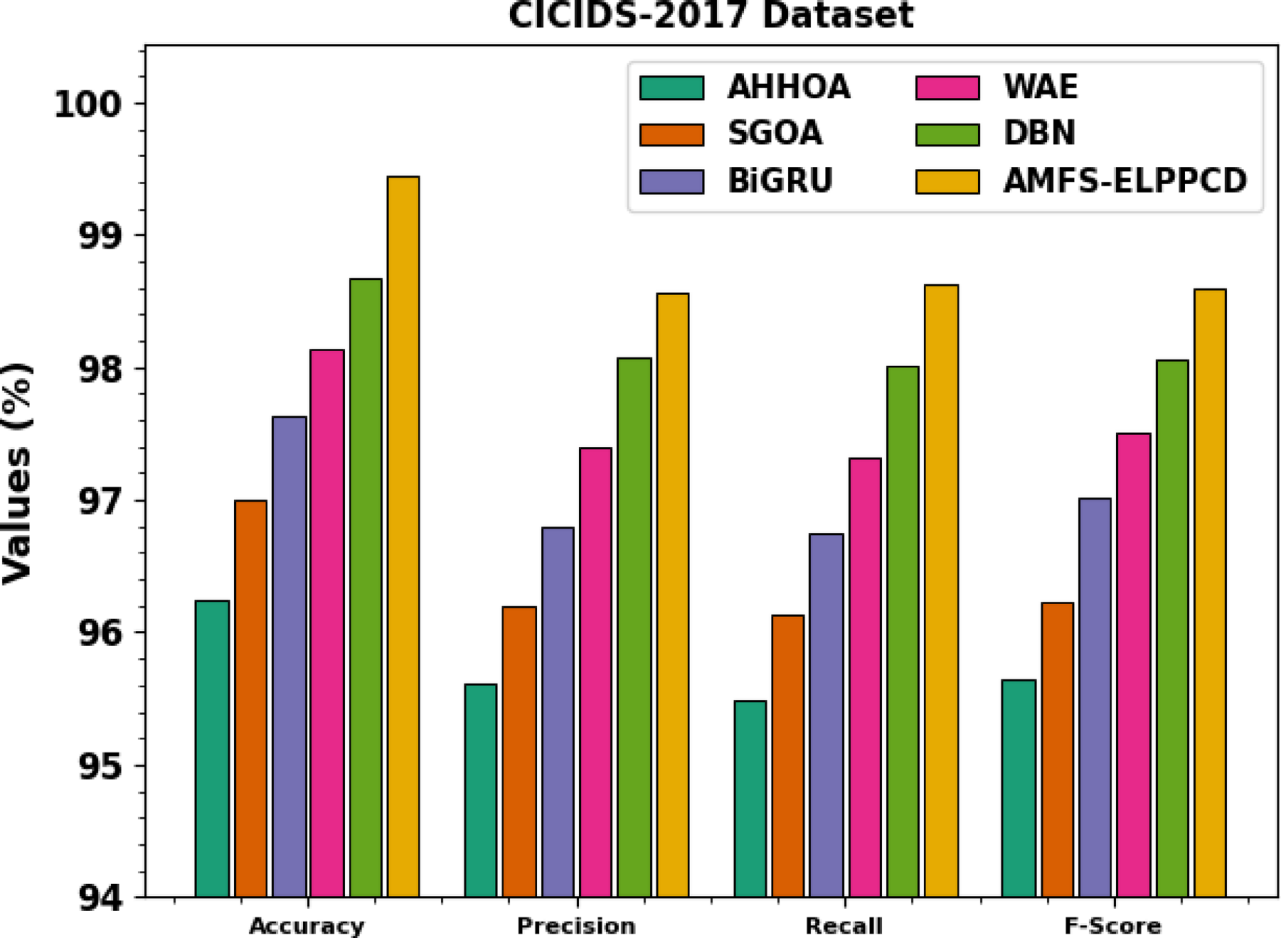
Ablation study of AMFS-ELPPCD technique under CICIDS-2017 dataset.

Table 6; Fig. 12 specify the ablation study of the AMFS-ELPPCD technique under the CICIDS-2017 dataset. The AHHOA model attains an $\:acc{u}_{y}$ of 96.24%, $\:pre{c}_{n}$ of 95.61%, recall of 95.48%, and an F-score of 95.65%. The SGOA model achieves an $\:acc{u}_{y}$ of 97.00%, $\:pre{c}_{n}$ of 96.20%, $\:rec{a}_{l}$ of 96.13%, and a $\:{F}_{score}$ of 96.23%. The BiGRU model achieves an $\:acc{u}_{y}$ of 97.63%, $\:pre{c}_{n}$ of 96.79%, $\:rec{a}_{l}$ of 96.75%, and a $\:{F}_{score}$ of 97.01%. The WAE model achieves an $\:acc{u}_{y}$ of 98.14%, $\:pre{c}_{n}$ of 97.39%, $\:rec{a}_{l}$ of 97.32%, and a $\:{F}_{score}$ of 97.51%. The DBN model achieves an $\:acc{u}_{y}$ of 98.67%, $\:pre{c}_{n}$ of 98.07%, $\:rec{a}_{l}$ of 98.01%, and a $\:{F}_{score}$ of 98.06%. Finally, the AMFS-ELPPCD model achieves an $\:acc{u}_{y}$ of 99.44%, $\:pre{c}_{n}$ of 98.57%, $\:rec{a}_{l}$ of 98.63%, and a $\:{F}_{score}$ of 98.60%.

**Table 6:** Ablation study of AMFS-ELPPCD technique under the CICIDS-2017 dataset.

**Model**	$\:\varvec{A}\varvec{c}\varvec{c}{\varvec{u}}_{\varvec{y}}$	$\:\varvec{P}\varvec{r}\varvec{e}{\varvec{c}}_{\varvec{n}}$	$\:\varvec{R}\varvec{e}\varvec{c}{\varvec{a}}_{\varvec{l}}$	$\:{\varvec{F}}_{\varvec{s}\varvec{c}\varvec{o}\varvec{r}\varvec{e}}$
CICIDS-2017 Dataset				
AHHOA	96.24	95.61	95.48	95.65
SGOA	97.00	96.20	96.13	96.23
BiGRU	97.63	96.79	96.75	97.01
WAE	98.14	97.39	97.32	97.51
DBN	98.67	98.07	98.01	98.06
AMFS-ELPPCD	99.44	98.57	98.63	98.60

Also, the proposed AMFS-ELPPCD method is studied under the NSLKDD dataset^47^. This dataset contains 50,000 samples under dual classes, such as normal and anomaly, as depicted in Table 7. The total number of features is 42, but only 25 have been selected.

**Table 7:** Details of the NSLKDD dataset.

Class	No. of Samples
*NSLKDD Dataset*	
Normal	20,000
Anomaly	20,000
Total Samples	50,000

**Fig. 13:**
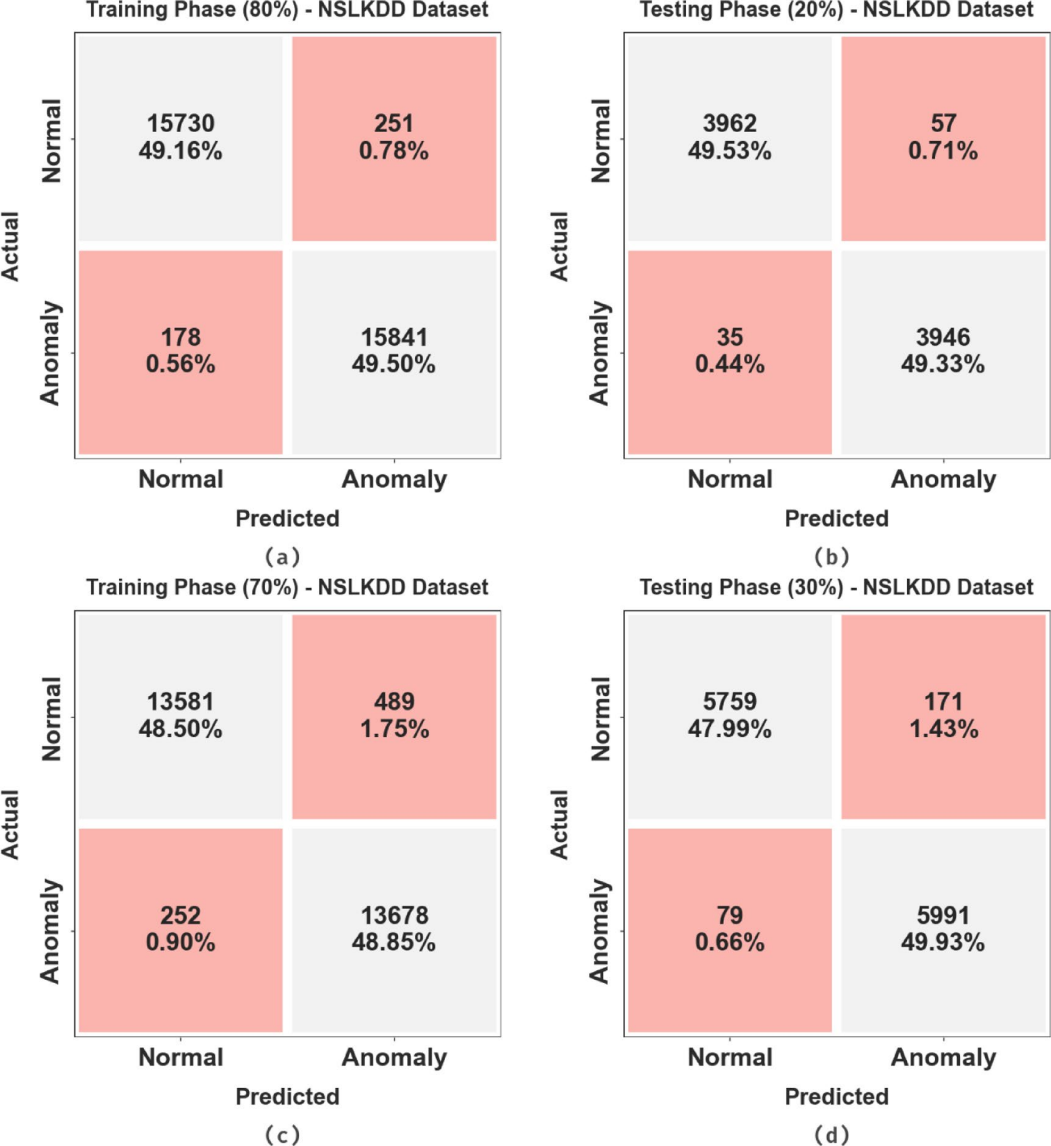
NSLKDD dataset (a-c) 80% and 70%TSPH and (b-d) 20% and 30%TSPH.

Figure 13 illustrates the confusion matrices of the AMFS-ELPPCD technique for the NSLKDD dataset during TRPH/TSPH depicting the classification performance between normal and anomaly classes. In the 80% TRPH, the model correctly classified 15,730 normal and 15,841 anomaly instances, with minimal misclassifications. During the 20% TSPH, it maintained similar accuracy, detecting 3962 normal and 3946 anomaly samples correctly. The 70% TRPH also illustrated robust performance, with 13,581 normal and 13,678 anomaly instances accurately predicted. For the 30% TSPH, the model exhibited consistent results, correctly classifying 5759 normal and 5991 anomaly instances. These results highlight the robustness and efficiency of the AMFS-ELPPCD approach in binary anomaly detection across diverse data splits.

**Fig. 14:**
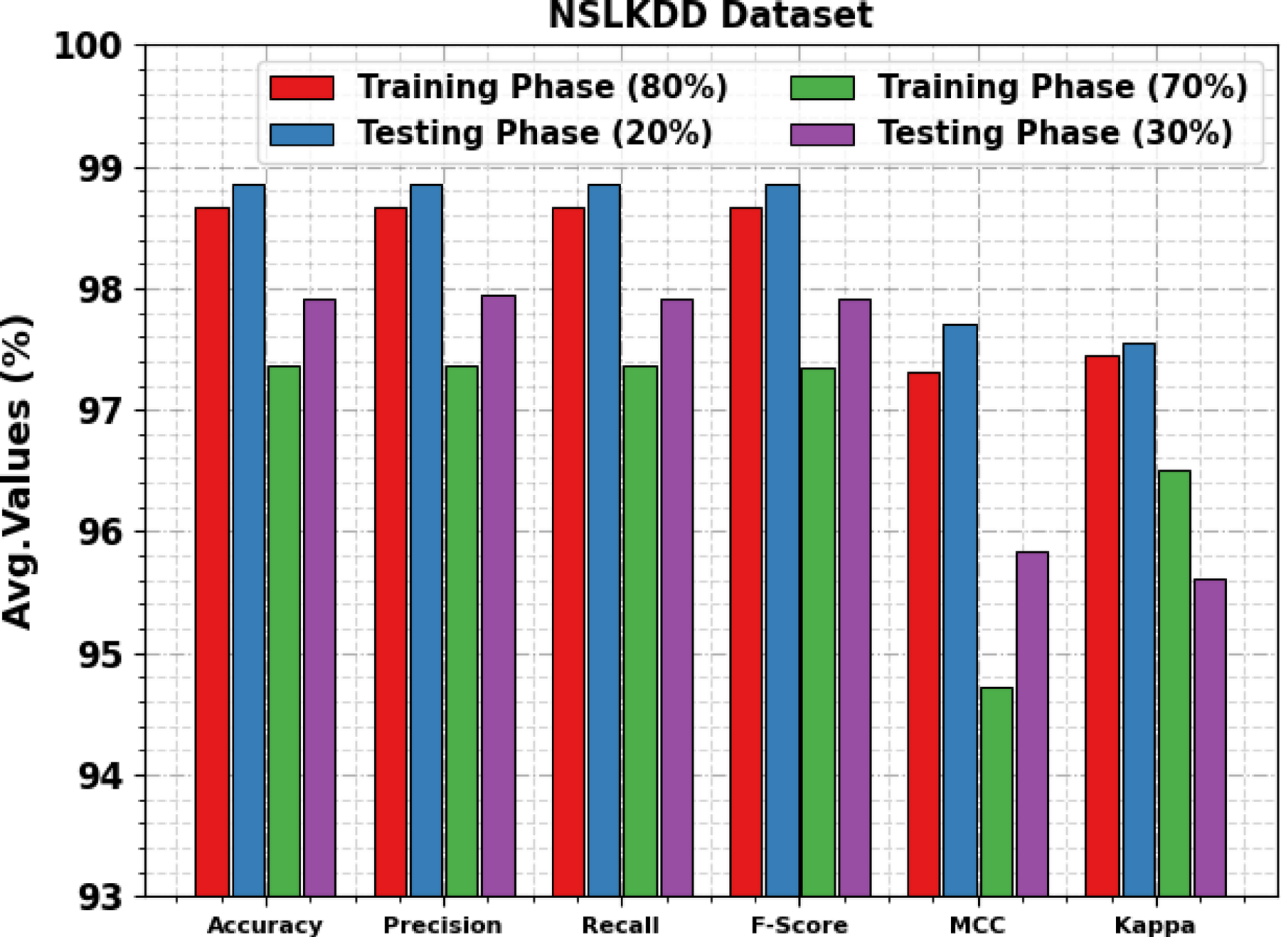
Average of AMFS-ELPPCD model on NSLKDD dataset.

Table 8; Fig. 14 illustrate the attack detection of the AMFS-ELPPCD method on the NSLKDD dataset. The performances suggest that the AMFS-ELPPCD method accurately recognized the samples below 80:20 and 70:30 TRPH/TSPH. Using 80%TRPH, the AMFS-ELPPCD approach attains an $\:acc{u}_{y}$, $\:pre{c}_{n}$, $\:rec{a}_{l}$, $\:{F}_{score}$, $\:MCC$, and Kappa of 98.66%, 98.66%, 98.66%, 98.66%, 97.32%, 97.45%, respectively. Also, using 20%TSPH, the AMFS-ELPPCD approach attains an $\:acc{u}_{y}$, $\:pre{c}_{n}$, $\:rec{a}_{l}$, $\:{F}_{score}$, $\:MCC$, and Kappa of 98.85%, 98.85%, 98.85%, 98.85%, 97.70%, and 97.55%, correspondingly. Moreover, based on 70%TSPH, the AMFS-ELPPCD approach attains an $\:acc{u}_{y}$, $\:pre{c}_{n}$, $\:rec{a}_{l}$, $\:{F}_{score}$, $\:MCC$, and Kappa of 97.36%, 97.36%, 97.36%, 97.35%, 94.72%, 96.51%, respectively. Besides, on 30%TSPH, the AMFS-ELPPCD approach attains an $\:acc{u}_{y}$, $\:pre{c}_{n}$, $\:rec{a}_{l}$, $\:{F}_{score}$, $\:MCC$, and Kappa of 97.91%, 97.94%, 97.91%, 97.92%, 95.84%, and 95.61%, respectively.

**Table 8:** Attack detection of AMFS-ELPPCD model on the NSLKDD dataset.

Classes	$\:\varvec{A}\varvec{c}\varvec{c}{\varvec{u}}_{\varvec{y}}$	$\:\varvec{P}\varvec{r}\varvec{e}{\varvec{c}}_{\varvec{n}}$	$\:\varvec{R}\varvec{e}\varvec{c}{\varvec{a}}_{\varvec{l}}$	$\:{\varvec{F}}_{\varvec{s}\varvec{c}\varvec{o}\varvec{r}\varvec{e}}$	$\:\varvec{M}\varvec{C}\varvec{C}$	Kappa
*TRPH (80%)*						
Normal	98.43	98.88	98.43	98.65	97.32	97.56
Anomaly	98.89	98.44	98.89	98.66	97.32	97.34
Average	98.66	98.66	98.66	98.66	97.32	97.45
*TSPH (20%)*						
Normal	98.58	99.12	98.58	98.85	97.70	97.98
Anomaly	99.12	98.58	99.12	98.85	97.70	97.12
Average	98.85	98.85	98.85	98.85	97.70	97.55
*TRPH (70%)*						
Normal	96.52	98.18	96.52	97.34	94.72	95.48
Anomaly	98.19	96.55	98.19	97.36	94.72	97.54
Average	97.36	97.36	97.36	97.35	94.72	96.51
*TSPH (30%)*						
Normal	97.12	98.65	97.12	97.88	95.84	96.23
Anomaly	98.70	97.22	98.70	97.96	95.84	94.98
Average	97.91	97.94	97.91	97.92	95.84	95.61

**Fig. 15:**
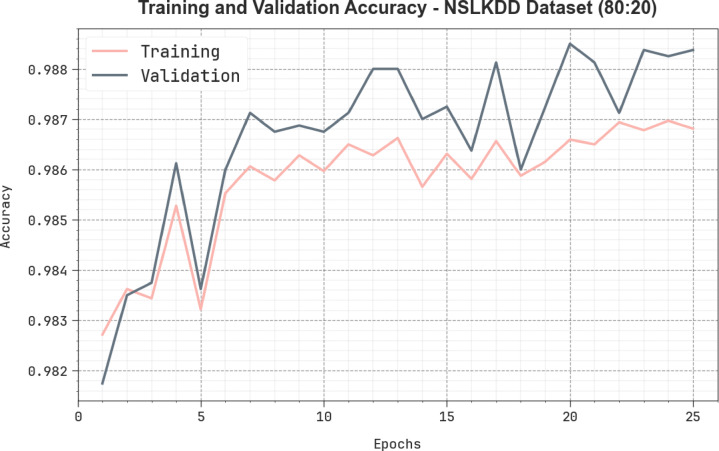
$\:Acc{u}_{y}$ curve of AMFS-ELPPCD method on NSLKDD dataset.

Figure 15 depicts the TRA $\:acc{u}_{y}$ and VAL $\:acc{u}_{y}$ solutions of the AMFS-ELPPCD technique on the NSLKDD dataset below 80%TRPH and 20%TSPH. The $\:acc{u}_{y}$ analysis is calculated across a period of 0–25 epochs. The figure underscored that the TRA and VAL $\:acc{u}_{y}$ analysis shows a cumulative trend, which informed the capacity of the AMFS-ELPPCD methodology using superior outcomes through multiple iterations. Simultaneously, TRA and VAL $\:acc{u}_{y}$ values remain closer across the epochs, which diminishes overfitting and expresses the optimal performance of the AMFS-ELPPCD methodology, which guarantees reliable calculation on unseen samples.

**Fig. 16:**
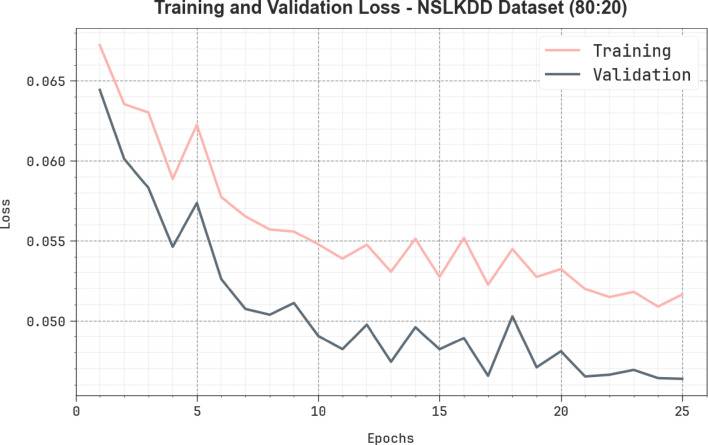
Loss analysis of AMFS-ELPPCD method on the NSLKDD dataset.

Figure 16 shows the TRA loss (TRALOS) and VAL loss (VALLOS) graph of the AMFS-ELPPCD method on the NSLKDD dataset below 80%TRPH and 20%TSPH. The loss values are calculated through a period of 0–25 epochs. It is exemplified that the values of TRALOS and VALLOS demonstrate a reducing trend, which identifies the ability of the AMFS-ELPPCD methodology to equalize an equilibrium among generalization and data fitting. The succeeding dilution in values of loss and securities results in a higher performance of the AMFS-ELPPCD methodology and tunes the calculation results gradually.

**Fig. 17:**
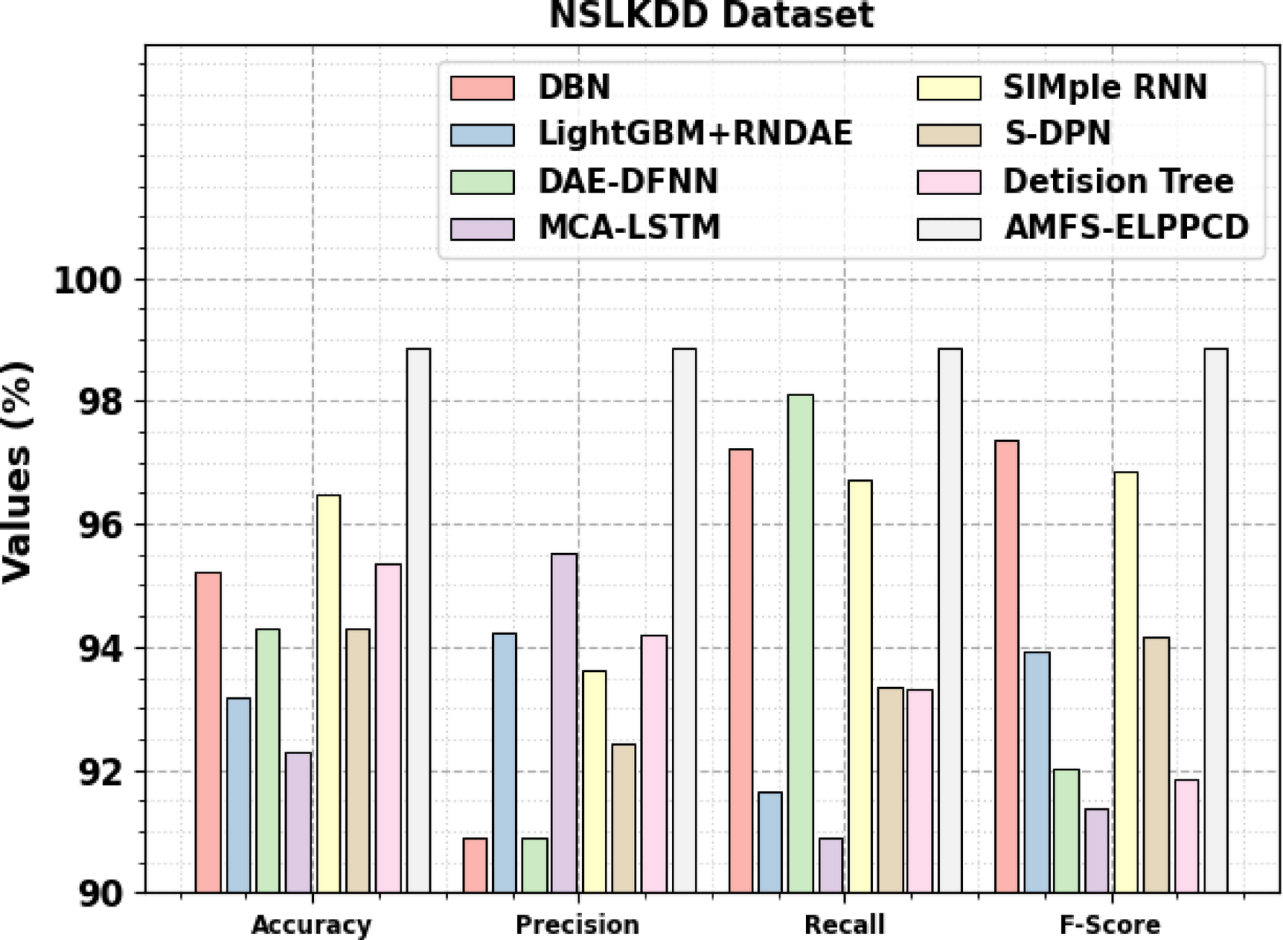
Comparative analysis of the AMFS-ELPPCD method on the NSLKDD dataset.

Table 9; Fig. 17 examine the comparative study of the AMFS-ELPPCD methodology on the NSLKDD dataset with existing approaches^48–52^. The performances underscored that the DBN, LightGBM + RNDAE, DAE-DFNN, MCA-LSTM, SIMple RNN, S-DPN, and DT models have stated poorer performance. While, the proposed AMFS-ELPPCD technique indicated maximum performance with improved $\:acc{u}_{y}$, $\:pre{c}_{n}$, $\:rec{a}_{l}$, and $\:{F}_{score}$ of 98.85%, 98.85%, 98.85%, and 98.85%, respectively.

**Table 9:** Comparative analysis of the AMFS-ELPPCD model on the NSLKDD dataset.

NSLKDD Dataset				
**Model**	$\:\varvec{A}\varvec{c}\varvec{c}{\varvec{u}}_{\varvec{y}}$	$\:\varvec{P}\varvec{r}\varvec{e}{\varvec{c}}_{\varvec{n}}$	$\:\varvec{R}\varvec{e}\varvec{c}{\varvec{a}}_{\varvec{l}}$	$\:{\varvec{F}}_{\varvec{s}\varvec{c}\varvec{o}\varvec{r}\varvec{e}}$
DBN	95.23	90.88	97.24	97.38
LightGBM + RNDAE	93.17	94.22	91.65	93.93
DAE-DFNN	94.30	90.89	98.11	92.01
MCA-LSTM	92.30	95.54	90.89	91.36
SIMple RNN	96.47	93.61	96.73	96.86
S-DPN	94.31	92.44	93.34	94.15
DT	95.36	94.20	93.30	91.84
AMFS-ELPPCD	98.85	98.85	98.85	98.85

**Fig. 18:**
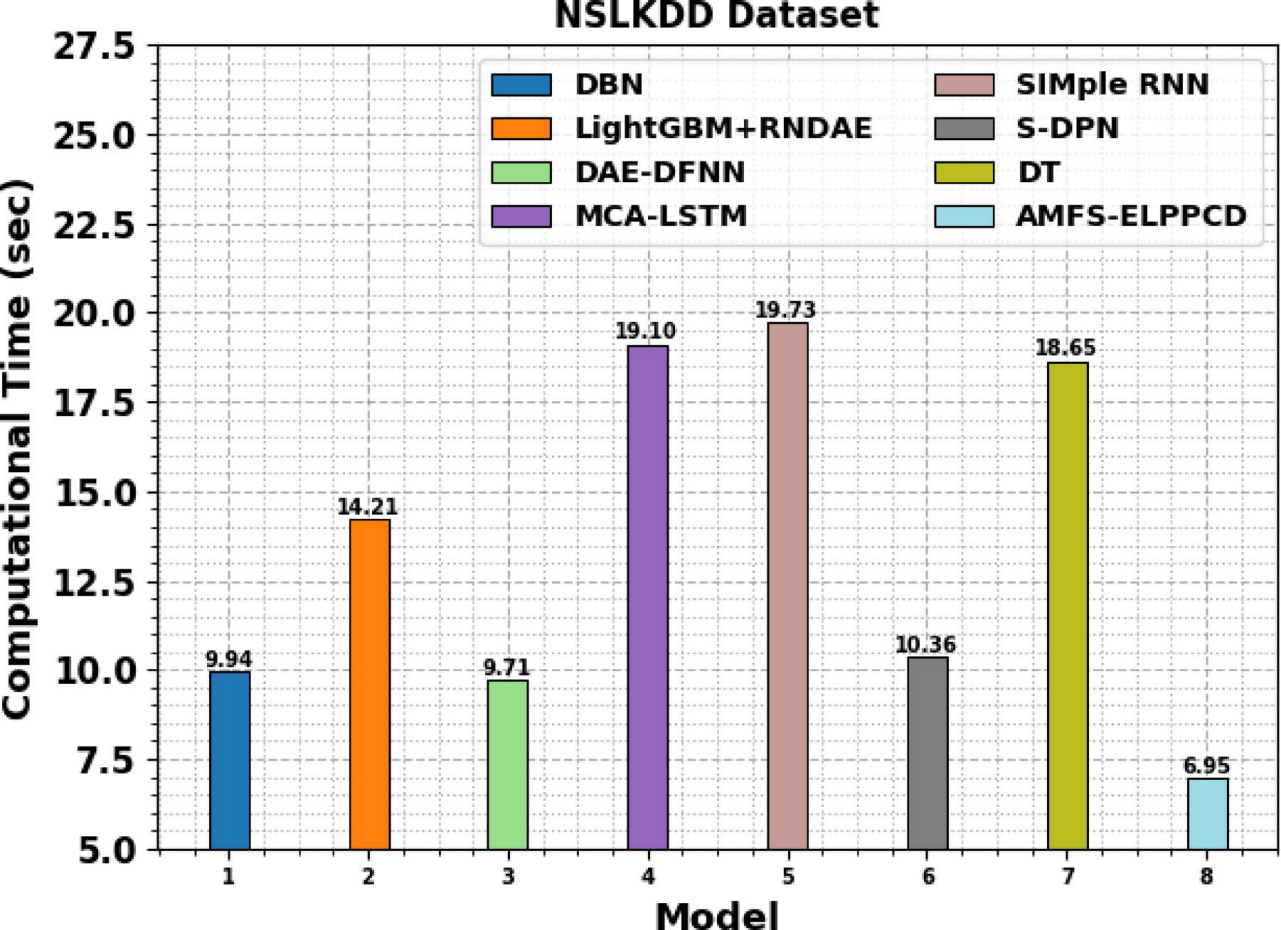
CT evaluation of AMFS-ELPPCD methodology with the existing techniques.

Table 10; Fig. 18 demonstrates the CT evaluation of the AMFS-ELPPCD methodology with the existing techniques. The AMFS-ELPPCD methodology achieved the lowest CT of 6.95 s, demonstrating significant performance in terms of speed. In comparison, DBN recorded a CT of 9.94 s, while DAE-DFNN performed slightly faster at 9.71 s. The S-DPN model required 10.36 s, and LightGBM combined with RNDAE took 14.21 s. More complex models such as MCA-LSTM and SIMple RNN exhibited the highest CTs of 19.10 and 19.73 s respectively. DT model also illustrated a higher CT of 18.65 s. These results emphasize the computational efficiency of the AMFS-ELPPCD model when applied to the NSLKDD dataset.

**Table 10:** CT evaluation of AMFS-ELPPCD methodology with the existing techniques.

NSLKDD Dataset	
Model	CT (sec)
DBN	9.94
LightGBM + RNDAE	14.21
DAE-DFNN	9.71
MCA-LSTM	19.10
SIMple RNN	19.73
S-DPN	10.36
DT	18.65
AMFS-ELPPCD	6.95

**Fig. 19:**
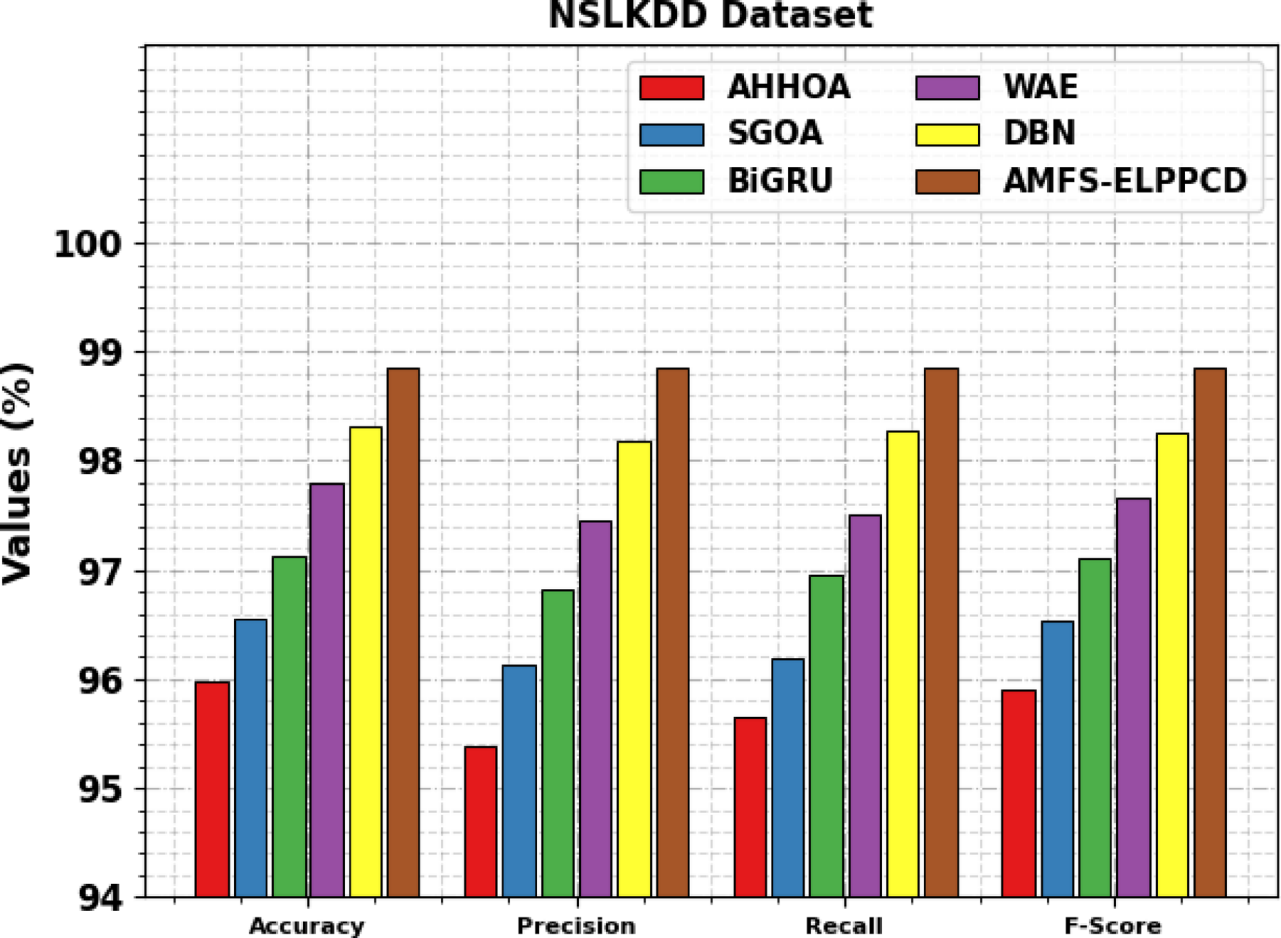
Ablation study of AMFS-ELPPCD approach under NSLKDD dataset.

Table 11; Fig. 19 demonstrate the ablation study of the AMFS-ELPPCD model under the NSLKDD dataset. The AHHOA model achieves an $\:acc{u}_{y}$ of 95.98%, $\:pre{c}_{n}$ of 95.39%, recall of 95.65%, and an F-score of 95.91%. The SGOA model achieves an $\:acc{u}_{y}$ of 96.56%, $\:pre{c}_{n}$ of 96.13%, $\:rec{a}_{l}$ of 96.19%, and a $\:{F}_{score}$ of 96.53%. The BiGRU model achieves an $\:acc{u}_{y}$ of 97.13%, $\:pre{c}_{n}$ of 96.83%, $\:rec{a}_{l}$ of 96.96%, and a $\:{F}_{score}$ of 97.11%. The WAE model achieves an $\:acc{u}_{y}$ of 97.79%, $\:pre{c}_{n}$ of 97.45%, $\:rec{a}_{l}$ of 97.51%, and a $\:{F}_{score}$ of 97.66%. The DBN model achieves an $\:acc{u}_{y}$ of 98.32%, $\:pre{c}_{n}$ of 98.19%, $\:rec{a}_{l}$ of 98.27%, and a $\:{F}_{score}$ of 98.25%. Finally, the AMFS-ELPPCD model achieves an $\:acc{u}_{y}$ of 98.85%, $\:pre{c}_{n}$ of 98.85%, $\:rec{a}_{l}$ of 98.85%, and a $\:{F}_{score}$ of 98.85%.

**Table 11:** Ablation study of AMFS-ELPPCD approach under NSLKDD dataset.

**Model**	$\:\varvec{A}\varvec{c}\varvec{c}{\varvec{u}}_{\varvec{y}}$	$\:\varvec{P}\varvec{r}\varvec{e}{\varvec{c}}_{\varvec{n}}$	$\:\varvec{R}\varvec{e}\varvec{c}{\varvec{a}}_{\varvec{l}}$	$\:{\varvec{F}}_{\varvec{s}\varvec{c}\varvec{o}\varvec{r}\varvec{e}}$
*NSLKDD Dataset*				
AHHOA	95.98	95.39	95.65	95.91
SGOA	96.56	96.13	96.19	96.53
BiGRU	97.13	96.83	96.96	97.11
WAE	97.79	97.45	97.51	97.66
DBN	98.32	98.19	98.27	98.25
AMFS-ELPPCD	98.85	98.85	98.85	98.85

Table 12 specifies the computational efficiency and memory usage of the AMFS-ELPPCD model. The computational efficiency of various DL models was assessed using two metrics: FLOPs in millions and GPU usage. Among all the evaluated methods, the AMFS-ELPPCD model illustrated the highest efficiency, requiring only 1.44 million FLOPs and consuming just 1.81 units of GPU resources. In contrast, conventional models such as VGG16 required significantly more computational power with 24.68 million FLOPs and 6.52 GPU usage. ResNet-50, although more efficient than VGG16, still required 5.50 million FLOPs and 3.85 GPU. MBNet and Fine-tuned MobileNetV2 exhibited moderate needs with 7.15 and 15.15 million FLOPs, and GPU usage of 3.08 and 3.38 respectively. The Contourlet CNN performed better in terms of lower computational requirements with 3.70 million FLOPs and 2.74 GPU usage. Overall, the results highlight the superior computational efficiency of the AMFS-ELPPCD model across both metrics.

**Table 12:** Comparison of computational efficiency and memory usage of AMFS-ELPPCD method.

Method	FLOPs (M)	GPU
VGG16	24.68	6.52
ResNet-50	5.50	3.85
MBNet	7.15	3.08
Fine-tune MobileNetV2	15.15	3.38
Contourlet CNN	3.70	2.74
AMFS-ELPPCD	1.44	1.81

## Conclusion and future work

In this manuscript, the AMFS-ELPPCD technique is proposed. The main aim of the AMFS-ELPPCD model is to detect and classify anomalous behaviour for cybersecurity attacks in IoT networks. The data normalization stage initially applies Z-score normalization to convert input data into a beneficial format. For the subset selection of the feature process, the AMFS-ELPPCD model utilizes AHHO. Furthermore, ensemble models such as BiGRU, WAE, and DBN are used in the classification process. Finally, the SGO optimally adjusts the ensemble classifiers’ hyperparameter values, resulting in more excellent classification performance. A set of simulations is performed to exhibit the promising results of the AMFS-ELPPCD under dual datasets. The experimental validation of the AMFS-ELPPCD technique portrayed a superior accuracy value of 99.44% and 98.85% under the CICIDS-2017 and NSLKDD datasets over existing models.

### Limitations and future works of the AMFS-ELPPCD model

The limitation of the AMFS-ELPPCD technique is its dependence on specific datasets, which may not fully represent real-world, various network environments, potentially affecting the generalization of the results. Furthermore, the models employed may not account for all attacks, specifically emerging or advanced cyberthreats. The computational complexity of specific techniques could restrict their applicability in resource-constrained devices or environments with limited computational power. Furthermore, the requirement for large-scale labelled datasets for training could present threats in dynamic and evolving network settings. Future works may concentrate on integrating adaptive models capable of learning from smaller, unlabeled datasets and enhancing the scalability and real-time performance of the proposed methods. Exploration of more lightweight models for resource-constrained environments and wide validation across diverse IoT scenarios would also be beneficial. Lastly, enhanced techniques for dealing with adversarial attacks in IoT networks should be explored to ensure greater robustness. As a future direction, the model may be extended and evaluated on more imbalanced and contemporary datasets such as TON_IoT and BoT-IoT to validate its robustness further.

## Data Availability

The data supporting this study’s findings are openly available at https://www.kaggle.com/datasets/chethuhn/network-intrusion-dataset and https://archive.ics.uci.edu/dataset/130/kdd+cup+1999+data, reference number [47, 48].
